# Afadin mediates cadherin-catenin complex clustering on F-actin linked to cooperative binding and filament curvature

**DOI:** 10.1126/sciadv.adu0989

**Published:** 2025-02-14

**Authors:** Rui Gong, Matthew J. Reynolds, Xiaoyu Sun, Gregory M. Alushin

**Affiliations:** Laboratory of Structural Biophysics and Mechanobiology, The Rockefeller University, New York, NY, USA.

## Abstract

The E-cadherin–β-catenin–αE-catenin (cadherin-catenin) complex couples the cytoskeletons of neighboring cells at adherens junctions (AJs) to mediate force transmission across epithelia. Mechanical force and auxiliary binding partners converge to stabilize the cadherin-catenin complex’s inherently weak binding to actin filaments (F-actin) through unclear mechanisms. Here, we show that afadin’s coiled-coil (CC) domain and vinculin synergistically enhance the cadherin-catenin complex’s F-actin engagement. The cryo–electron microscopy (cryo-EM) structure of an E-cadherin–β-catenin–αE-catenin–vinculin–afadin-CC supra-complex bound to F-actin reveals that afadin-CC bridges adjacent αE-catenin actin-binding domains along the filament, stabilizing flexible αE-catenin segments implicated in mechanical regulation. These cooperative binding contacts promote the formation of supra-complex clusters along F-actin. Additionally, cryo-EM variability analysis links supra-complex binding along individual F-actin strands to nanoscale filament curvature, a deformation mode associated with cytoskeletal forces. Collectively, this work elucidates a mechanistic framework by which vinculin and afadin tune cadherin-catenin complex–cytoskeleton coupling to support AJ function across varying mechanical regimes.

## Teaser

Afadin strengthens adherens junctions by stabilizing cadherin-catenin complex clusters on curved F-actin.

## INTRODUCTION

Adherens junctions (AJs) are cell-cell adhesion complexes that transmit mechanical signals across epithelia to coordinate multicellular dynamics during development and tissue homeostasis (*1*–*3*). AJ dysregulation leads to loss of cell-cell contact and epithelial-to-mesenchymal transition, hallmarks of cancer metastasis (*4*, *5*). An interconnected protein network modulates the coupling strength between AJs and cytoskeletal actin filaments (F-actin) to support AJ function across the wide range of force regimes encountered in tissues (*2*, *6*). The central component of this network is the heterotrimeric cadherin-catenin complex consisting of E-cadherin, β-catenin, and αE-catenin. E-cadherin is a transmembrane protein that directly mediates cell-cell contacts. The E-cadherin extracellular domains of two neighboring cells directly bind each other in trans, while their intracellular domains interact with β-catenin, which, in turn, associates with the actin-binding protein αE-catenin (*6*, *7*). Multiple cadherin-catenin complexes further assemble into clusters within an AJ, mediated by cis interactions between E-cadherin extracellular domains within the same plasma membrane that are reinforced by cooperative binding of αΕ-catenin to cytoplasmic F-actin (*8*, *9*). This multilayered network is critical for AJs to form mechanically robust connections between cells, yet it remains unclear how the interactions between its components are dynamically tuned to transmit and respond to cytoskeletal forces.

Force enhances F-actin binding by the cadherin-catenin complex, as well as its interactions with additional AJ proteins, to promote adhesion and facilitate mechanical signal transduction at AJs (*10*). The cadherin-catenin complex in isolation is autoinhibited, displaying weak intrinsic binding affinity for F-actin and other adhesion partners (*11*–*13*). Mechanical regulation is focused on αΕ-catenin, whose C-terminal actin-binding domain (ABD) and central M domain undergo structural transitions associated with binding-partner engagement that can be modulated by force (*14*–*16*). The complex forms a mechanically stabilized catch bond with F-actin in the presence of forces on the order of 10 pN. Mechanistically, this has been attributed to force-stabilized displacement of the first two α helices (H0-H1) from the αΕ-catenin ABD helical bundle, facilitating a torsional rearrangement of the bundle concomitant with strong F-actin binding (*16*, *17*). Additionally, lower magnitude forces (~1 pN) applied solely across F-actin enhance binding by the isolated αΕ-catenin ABD, which is mediated by the protein’s flexible 35–amino acid C-terminal extension (CTE) (*18*). Tension furthermore promotes unfurling of the αΕ-catenin M domain, facilitating binding by the αΕ-catenin structural homolog vinculin (*14*). Vinculin engages the M1 subdomain through its N-terminal “head” domain, facilitating F-actin binding and catch bonding by its C-terminal “tail” ABD to reinforce cadherin-catenin complex–cytoskeleton coupling (*11*, *13*, *19*). Additionally, the isolated vinculin head domain enhances the cadherin-catenin complex’s cooperative F-actin binding in the presence of mechanical load (*20*). Despite substantial progress in establishing the biophysical mechanisms of this core network of force-modulated interactions, it remains largely unknown how the cadherin-catenin complex interfaces with additional critical adhesion proteins to maintain AJ mechanical integrity and function.

One such factor is afadin, a large (206 kDa) multidomain scaffolding protein with numerous AJ binding partners, including αΕ-catenin and F-actin (*21*, *22*). Afadin knockout is embryonically lethal in the mouse, where defective cell-cell junctions fail to support development (*23*, *24*). In human patients, dysregulated afadin expression has been associated with carcinogenesis and cancer metastasis in multiple tissue contexts, and somatic afadin loss-of-function mutations have recently been reported to drive a class of E-cadherin–positive metastatic breast cancer (*25*). Afadin inactivation in cultured epithelial cells delays initial AJ formation without impacting the integrity of mature adhesions (*26*, *27*), mirroring the mild phenotype of knocking out the *Drosophila* afadin homolog canoe in morphogenetically inactive tissues (*28*). However, during periods of tissue rearrangement featuring elevated cellular contractility such as morphogenesis, afadin or canoe ablation causes defective AJ remodeling, impairing processes including apical constriction (*28*, *29*), convergent extension (*30*, *31*), wound healing (*27*), and collective cell migration (*32*). This supports a key role for afadin in reinforcing AJs under conditions of high tissue tension.

Although it is structurally divergent from vinculin, afadin features both αΕ-catenin and F-actin binding domains. This suggests that it might function in a conceptually analogous fashion to vinculin by reinforcing the interaction between the cadherin-catenin complex and F-actin (*21*, *22*). Initially, the F-actin binding region (FAB) of afadin was assigned to its very C terminus (*22*, *29*). However, no experimental data have confirmed a direct afadin–F-actin interaction mediated by this region, and this segment in canoe is dispensable for *Drosophila* morphogenesis (*33*). Subsequently, an additional FAB, spanning residues 1514 to 1682, was identified using in vitro co-sedimentation assays (*34*). This region is located within a long intrinsically disordered region (IDR) flanked by the PDZ domain and the previously designated FAB. The IDR in canoe has been shown to be required for morphogenesis (*35*). Nevertheless, the relatively weak interaction between afadin and F-actin, with a dissociation constant of 3.8 ± 0.5 μM (*34*), challenges the model of afadin-mediated AJ stabilization occurring through a mechanism analogous to vinculin’s. Recently, afadin’s short coiled-coil region (residues 1393 to 1602, hereafter referred to as “afadin-CC”) within the IDR has been reported to enhance F-actin binding by the cadherin-catenin complex through an unknown mechanism, providing an alternative explanation for afadin’s stabilizing effect (*29*). Notably, afadin-CC includes an αE-catenin binding fragment (residues 1393 to 1453) that is predicted to form an α helix to interact with αE-catenin’s M3 domain (*35*, *36*). Although both vinculin and afadin contribute to AJ formation and remodeling, it remains unclear how they coordinately tune the coupling strength between the cadherin-catenin complex and F-actin to adapt to various force conditions.

Here, we show that vinculin and afadin collaborate to promote a highly activated form of the cadherin-catenin complex, with afadin-CC physically engaging mechanically regulated structural elements in αΕ-catenin to mediate cooperative F-actin binding. In biochemical assays, we find that vinculin and afadin-CC synergistically enhance the cadherin-catenin complex’s F-actin binding affinity. A 3.1-Å resolution cryo–electron microscopy (cryo-EM) structure of the five-component mammalian E-cadherin–β-catenin–αΕ-catenin–vinculin–afadin-CC supra-complex bound to F-actin resolves extensive contacts between afadin-CC and the αΕ-catenin ABD. Afadin-CC directly promotes cooperativity by bridging two adjacent ABDs, binding and stabilizing both the displaced helix H1 and the CTE. This induces folding of the entire CTE on the F-actin surface, which mediates additional F-actin and intra-ABD contacts, resulting in an extensive interaction network that tightly anchors neighboring cadherin-catenin complexes. Consistently, total internal reflection fluorescence (TIRF) studies show that the afadin-CC substantially enhances the cadherin-catenin complex’s cooperative F-actin binding, promoting the formation of supra-complex clusters along filaments. Furthermore, ablation of specific afadin-CC–αΕ-catenin ABD contacts eliminates afadin-CC’s stimulation of cadherin-catenin complex F-actin binding in vitro and disrupts AJ organization in cultured cells. From our cryo-EM dataset, we also obtained a structure featuring the supra-complex asymmetrically bound along one F-actin strand, which variability analysis links to F-actin curvature. This suggests afadin’s enhancement of cooperative F-actin binding by the cadherin-catenin complex is likely additionally promoted by nanoscale mechanical deformation of F-actin by cytoskeletal forces. Collectively, these data support a model in which afadin enhances the cadherin-catenin complex’s cytoskeletal engagement by stabilizing a network of binding interactions on the F-actin surface, simultaneously bolstering the effects of mechanical force and binding partners such as vinculin.

## RESULTS

### Reciprocally stimulated F-actin binding by the cadherin-catenin complex and vinculin

**Fig. 1.**
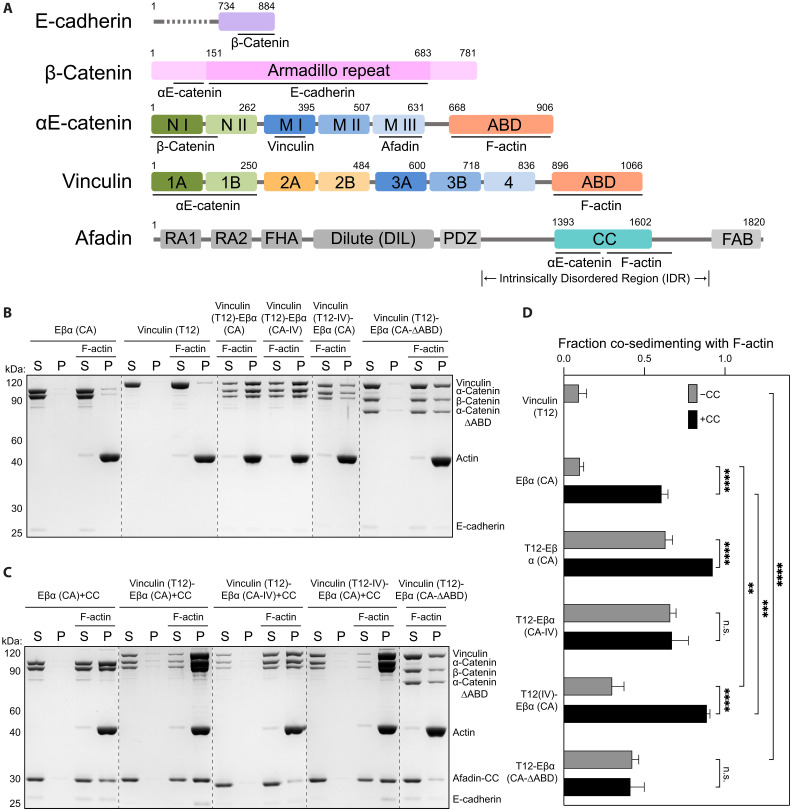
Vinculin and afadin synergistically enhance the cadherin-catenin complex’s F-actin binding. (**A**) Domain organization of E-cadherin, β-catenin, αE-catenin, vinculin, and afadin. Domains used in this study are colored and sequence boundaries are indicated. ABD, actin-binding domain; CC, coiled coil; FAB, F-actin binding. (**B** to **C**) Co-sedimentation assays showing the binding of indicated proteins and protein complexes to F-actin. S, supernatant; P, pellet. (**D**) Quantification of the fraction of indicated proteins or protein complexes that co-sedimented with F-actin in the pellet. Measurements were corrected by subtracting the amount of protein which sedimented in the absence of F-actin. Data are presented as means ± SD of three independent experiments. Conditions were compared using two-way analysis of variance (ANOVA) with Tukey’s multiple comparison test: Not significant (n.s.), *P* ≥ 0.05; **P < 0.01; ***P < 0.001; ****P < 0.0001. Dotted lines indicate stitching interfaces between gels.

To probe how the interplay of multiple actin-binding adhesion proteins can modulate AJ-cytoskeleton coupling strength, we first examined the cadherin-catenin complex and vinculin in F-actin co-sedimentation assays. In isolation, both factors have been reported to adopt a closed conformation, displaying minimal binding to each other and to F-actin (*12*, *15*, *37*). Consistently, we find that wild-type (WT) full-length vinculin and a minimal soluble ternary cadherin-cadherin complex (hereafter “Eβα”), which includes the intracellular β-catenin binding region of E-cadherin, full-length β-catenin, and full-length αE-catenin, each individually exhibited weak F-actin binding (Fig. 1A and fig. S1, A and D). To test whether binding between vinculin and the cadherin-catenin complex mutually influences their F-actin engagement, we introduced mutations previously reported to reduce autoinhibitory interactions in vinculin (*38*) [D974A/K975A/R976A/R978A, “vinculin(T12)”] and αE-catenin (*39*) [M319G/R326E/R551E, “α(CA)”] in the context of the minimal Eβα trimer. Unexpectedly, under our assay conditions both mutants still displayed weak F-actin binding in isolation, comparable to that of the respective WT proteins, implying that their ABDs are still substantially autoinhibited (Fig. 1, B and D). However, we find that the mutant proteins strongly interact, allowing us to reconstitute a stoichiometric Eβα(CA)-vinculin(T12) tetramer that exhibited substantially increased F-actin binding (Fig. 1, B and D, and fig. S1E).

We next assessed the contributions of the vinculin and αΕ-catenin ABDs to this enhanced F-actin binding activity. We first validated point mutations previously reported to reduce F-actin binding in vinculin [I997A/V1001A, “vinculin (IV)”] and αE-catenin [I792A/V796A, “α(IV)”] by examining their isolated ABDs (*40*, *41*). Consistent with previous studies, the IV mutations reduced F-actin binding by the vinculin ABD to undetectable levels in our assay and substantially attenuated F-actin binding by the αΕ-catenin ABD (fig. S1, C and D). We then examined these mutants in the context of the higher-order complex. The IV mutations in vinculin [Eβα(CA)–vinculin(T12-IV)] markedly reduced F-actin binding of the tetramer, which, nevertheless, bound better than the minimal cadherin-catenin complex trimer [Eβα(CA); Fig. 1, B and C]. Conversely, these mutations in αΕ-catenin [Eβα(CA-IV)–vinculin(T12)] had only a marginal impact on the tetramer’s F-actin engagement (Fig. 1, B and C). As the IV mutations reduced but did not eliminate actin-binding by the αΕ-catenin ABD, we also examined a complex where this domain was completely deleted [Eβα(CA-ΔABD)–vinculin(T12)]. Removing the αΕ-catenin ABD substantially attenuated F-actin binding, yet this complex nevertheless bound more tightly than vinculin(T12) (Fig. 1, B and C). Collectively, these data show that vinculin and the cadherin-catenin complex reciprocally enhance each other’s F-actin engagement. As the vinculin–cadherin-catenin complex interaction is known to occur through the vinculin Vh domain–αΕ-catenin M1 domain interface (Fig. 1A) (*15*), enhanced F-actin binding is likely mediated by an allosteric effect that releases both ABDs. While we find that the vinculin ABD is the primary actin-binding element of this complex, consistent with a recent report (*42*), our data suggest that the αΕ-catenin ABD also contributes to its overall F-actin engagement.

### Synergistically enhanced cadherin-catenin complex F-actin binding by afadin and vinculin

We next examined the effects of afadin on our reconstituted AJ complexes. The afadin-CC domain has previously been shown to enhance the F-actin binding of a chimerically fused β-catenin–αE-catenin dimer (*29*). Consistently, we find that the afadin-CC also markedly stimulates F-actin binding by the minimal constitutively active trimeric complex [Eβα(CA); Fig. 1, C and D]. However, this effect was absent for the WT trimer (Eβα), and a trimer featuring IV mutations in the αΕ-catenin ABD [Eβα(CA-IV); fig. S1, B and D], suggesting that afadin-CC stimulates αΕ-catenin ABD release to potentiate its F-actin binding. Consistent with this model, pulldown assays with αE-catenin fragments confirm a strong interaction specifically between afadin-CC and the M3 domain of αE-catenin (fig. S2A) (*36*). The distinct binding sites of vinculin (M1) and afadin (M3) on αE-catenin prompted us to investigate whether they can coincidentally engage the cadherin-catenin complex. In pulldown assays, we find that afadin-CC and activated vinculin simultaneously bind the minimal constitutively active trimer to form a stable pentameric complex [Eβα(CA)–vinculin(T12)–afadin-CC; fig. S2, B and C]. This pentamer binds F-actin even more robustly than the tetrameric vinculin–cadherin-catenin complex [Eβα(CA)–vinculin(T12); Fig. 1, C and D], indicating that vinculin and afadin can synergize to enhance the cadherin-catenin complex’s F-actin engagement.

In notable contrast to the tetrameric cadherin-catenin-vinculin complex [Eβα(CA)–vinculin(T12-IV)], disrupting vinculin’s F-actin binding activity had minimal impact on the stimulating effect of afadin-CC in the pentameric complex [Eβα(CA)–vinculin(T12-IV)–afadin-CC; Fig. 1, C and D], suggesting that this assembly instead primarily engages F-actin through the αE-catenin ABD. Moreover, vinculin featuring IV mutations [Eβα(CA)–vinculin(T12-IV)–afadin-CC] still stimulated the cadherin-catenin complex’s F-actin binding to a greater degree than afadin-CC alone [Eβα(CA)–afadin-CC; Fig. 1, C and D], indicating that afadin and vinculin converge to enhance F-actin binding through αE-catenin in the context of the pentameric complex. Consistently, complexes featuring lesions to the αE-catenin ABD [either IV mutations, Eβα(CA-IV)–vinculin(T12)–afadin-CC, or ABD deletion, Eβα(CA-ΔABD)–vinculin(T12)–afadin-CC] displayed substantial reductions in F-actin binding (Fig. 1, C and D). However, these complexes, nevertheless, bound F-actin to a similar degree as the corresponding tetrameric complexes in the absence of afadin-CC [Eβα(CA-IV)–vinculin(T12)/Eβα(CA-ΔABD)–vinculin(T12); Fig. 1, C and D], indicating that the vinculin ABD remains active in the pentamer, albeit making a diminished contribution to overall F-actin engagement. Collectively, these data indicate that afadin-CC and vinculin can simultaneously bind the cadherin-catenin complex to synergistically enhance its cytoskeletal association, primarily by stimulating F-actin binding through the αE-catenin ABD.

### Structure of a cadherin-catenin–vinculin–afadin-CC supra-complex bound to F-actin

**Fig. 2.**
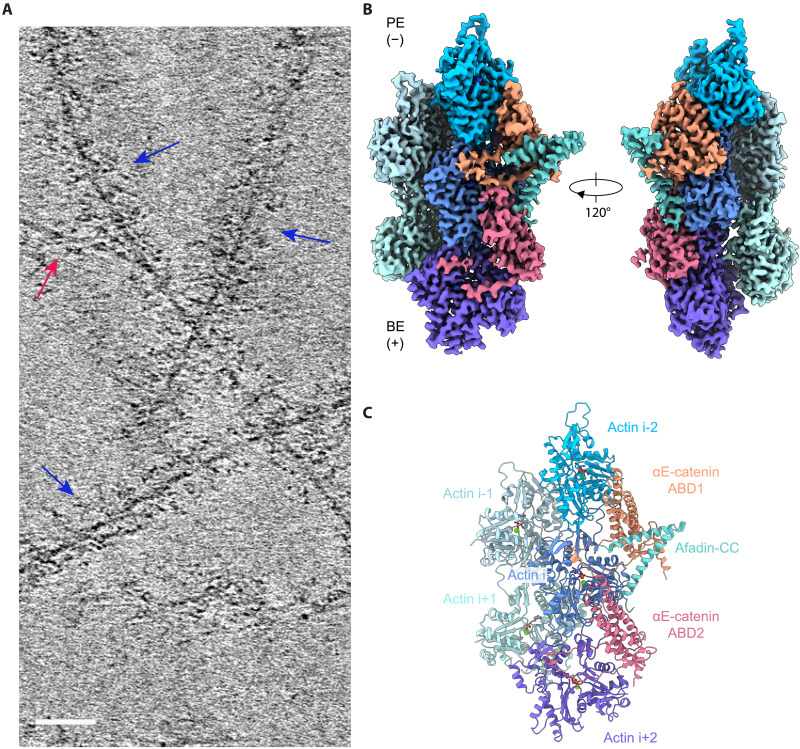
Cryo-EM structure of pentamer bound F-actin, visualizing αE-catenin ABD stabilization by afadin-CC. (**A**) Slice from cryo–electron tomogram highlighting pentameric complexes flexibly protruding from F-actin. Red and blue arrows indicate bare and pentamer bound F-actin, respectively. Scale bar, 50 nm. (**B**) Cryo-EM density map (3.1-Å resolution), which resolves two αE-catenin ABDs and one afadin-CC bound to F-actin. BE, barbed end; PE, pointed end. (**C**) Ribbon representation of the associated atomic model.

To investigate the mechanisms by which AJ partners enhance the cadherin-catenin complex’s F-actin binding, we performed cryo-EM structural studies. To specifically probe the regulatory roles of vinculin and afadin on αE-catenin’s F-actin binding activity, we investigated the pentameric complex featuring IV mutations in vinculin [Eβα(CA)-vinculin(T12-IV)-afadin-CC] bound to F-actin to minimize heterogeneity at the F-actin interface and potential confounding effects of the vinculin ABD. Previous studies have suggested a highly flexible linker between the αE-catenin ABD and the M domain (*43*, *44*). Consistently, in cryo–electron tomograms, we observed extended fibrous densities connected to actin filaments (Fig. 2A and movie S1), consistent with the 344-kDa mass of the complex, which appear to have no fixed orientation relative to the filament axis. Nevertheless, we pursued single-particle cryo-EM, reasoning that we might observe modulation of the αE-catenin ABD–F-actin interface that contributes to enhanced binding in the presence of afadin-CC.

Despite a substantial excess of pentamer (2.5 μM) versus F-actin (0.6 μM), we observed only partial decoration (Fig. 2A and fig. S3A), potentially due to crowding effects imposed by the large flexible mass of the complex. This, along with the strong signal from these flexible regions in the raw micrographs, presented a challenge for traditional template-based particle picking. We therefore used a neural network–based approach (Materials and Methods and fig. S3). Following two-dimensional (2D) classification, we observed only a small additional ordered density adjacent to F-actin (fig. S3C), consistent with our tomography studies, suggesting that the vast majority of the complex is flexibly tethered to the filament (movie S1). Two distinct categories of 2D classes included this extra density. One features straight F-actin with decoration along both strands (fig. S3F), consistent with prior structural studies of the isolated αE-catenin ABD bound to F-actin (*18*, *45*), while the other, which accounts for ~21% of particles in all high-quality 2D classes, unexpectedly features curved F-actin with decoration along only one strand (fig. S3N). The presence of this substantial curved subpopulation suggests a potential link between complex binding and F-actin remodeling.

To obtain a detailed view of the F-actin binding interface, we first focused on the well-ordered straight filament classes, which we reasoned would yield the highest-resolution 3D reconstruction, before analyzing the curved classes. Analysis of these segments produced a final density map at 3.1-Å resolution (Materials and Methods; Fig. 2B; fig. S4, A and B; and table S1). While this map predominantly resembled previously reported reconstructions of the isolated αE-catenin ABD bound F-actin (*18*, *45*), it featured three additional densities. The first is a coiled-coil density bridging two adjacent αE-catenin ABDs, which we identified as residues 1508 to 1578 of the afadin-CC using an AlphaFold2-predicted structure of this segment (fig. S4, C to E) (*46*). The second density extended from the previously resolved proximal portion of αE-catenin’s CTE (residues 844 to 871, which we here refer to as “CTE-N”), indicating folding of the disordered distal portion of the CTE (“CTE-C,” residues 872 to 906; fig. S4, C to E). High-quality density in two regions (residues 881 to 884 and 891 to 903) enabled us to model the entire CTE except for the final three residues (fig. S4E). The third and final extra density, a helical segment tucked alongside the afadin coiled-coil, was unambiguously assigned as residues 690 to 706 (the C-terminal half of H1) of the αE-catenin ABD, owing to its connection to H2 (fig. S4, C to E). Our final refined atomic model contains the entire sequence of actin except for the first four residues, residues 690 to 903 of αE-catenin and residues 1508 to 1578 of afadin.

In the structure, each αE-catenin ABD interacts with two neighboring actin subunits along the same strand, while each actin subunit contacts two adjacent ABDs (Fig. 2C). The two arms of the “V”-shaped afadin coiled-coil point toward the central axis of the filament, with each arm engaging one of the two neighboring ABDs. At each interface, the C-terminal arm of the coiled-coil makes extensive contacts with the ABD toward the minus (“pointed”) end of the filament (ABD1), while the N-terminal arm makes more limited contacts with the ABD toward the plus (“barbed”) end (ABD2; Fig. 2C).

### αE-catenin ABD force-sensitive flexible elements stabilized by afadin

**Fig. 3.**
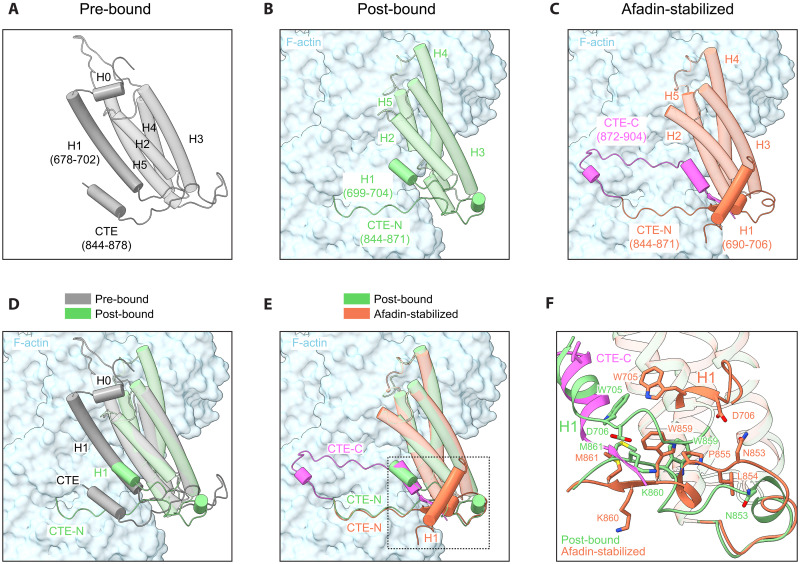
Structural remodeling of the αE-catenin ABD evoked by afadin-CC. (**A** to **C**) Structures of the αE-catenin ABD in three different conformational states. αE-catenin and F-actin are depicted in cartoon and surface representation, respectively. Pre-bound, crystal structure of αE-catenin (PDB: 4IGG, chain B); post-bound, cryo-EM structure of αE-catenin ABD alone bound to F-actin (PDB: 6UPV); afadin-stabilized, this study. The refolded CTE-C is highlighted in magenta. (**D**) Superposition of the αE-catenin ABD in the pre-bound and post-bound states. (**E**) Superposition of the αE-catenin ABD in the post-bound and afadin-stabilized states. (**F**) Detail view [boxed in (E)] highlighting αE-catenin ABD H1 and CTE structural rearrangements evoked by afadin-CC engagement.

Previous structural studies of the αE-catenin ABD have shown that substantial conformational remodeling accompanies its F-actin engagement (*18*, *45*). This includes an order-to-disorder transition of H0-H1, a torsional repacking of the H2-H5 helical bundle, and a shift of the proximal CTE-N, which collectively facilitate high-affinity binding to the filament (Fig. 3, A, B, and D, and movie S2). Functional studies have suggested that H0-H1 release is promoted by tension to mediate catch-bond formation (*17*, *40*), while CTE-C is required for force-activated F-actin binding when load is applied across the filament (*18*). Superimposing the αΕ-catenin ABD from our supra-complex structure on our previous structure of the isolated ABD bound to F-actin shows nearly identical conformations of H2-H5 [residues 707 to 843, root mean square deviation (RMSD) of 0.466 Å for 118 aligned Cα atoms] and the FAB of CTE-N (residues 863 to 871, RMSD of 0.290 Å for 9 aligned Cα atoms; Fig. 3E). Modulation of this interface is therefore unlikely to contribute to the supra-complex’s enhanced F-actin affinity. However, the afadin coiled-coil induces substantial remodeling of H1, reconfiguration of the proximal region of CTE-N, and the complete refolding of CTE-C to mediate specific F-actin and intra-ABD contacts, suggesting that afadin’s engagement of these flexible elements potently stabilizes F-actin binding (Fig. 3, C and E).

In previous structures of the isolated αE-catenin ABD in the absence of F-actin (the “pre-bound” state), the fully folded H1 packs against H2 and H5 (Fig. 3A and movie S2) (*44*). In structures of the isolated αE-catenin ABD bound to F-actin, a short stub of H1 remains docked on the helical bundle upon F-actin binding (the “post-bound” state; Fig. 3B and movie S2). In notable contrast, H1 is fully displaced in the afadin-stabilized state (Fig. 3C and movie S3). H1 from ABD1 is repositioned to bind beneath the central joint of the afadin coiled-coil, where residues 694 to 698 become reordered, forming interactions that mutually stabilize afadin and αΕ-catenin. Furthermore, residues W705 and D706 immediately C-terminal to H1 rearrange, incorporating to extend the helix (Fig. 3, C and F).

While CTE-C was unresolved in both the pre-bound and post-bound states of the isolated αE-catenin ABD, afadin notably stabilizes refolding of the entire αE-catenin CTE on the F-actin surface, pinioning it beneath the C-terminal arm of the coiled-coil (Fig. 3C, fig. S4D, and movie S3). The ordered CTE-C of ABD1 first extends upward through a flexible linker (residues 872 to 880), followed by a patch of well-resolved residues (881 to 884), which interface with subdomain 4 of actin i. It then turns toward the H2-H5 helical bundle of ABD1 through another linker (residues 885 to 892). The C-terminal residues 893 to 898 refold into a short α helix, which sits in a groove vacated by complete displacement of H1 (Fig. 3, E and F). This is followed by a short β strand (residues 900 to 902), which inserts into a pocket generated by remodeling of residues 859 to 861, where it is surrounded by the C-terminal coil of afadin-CC, as well as H1, H2, H5, and CTE-N of ABD1 (Fig. 3, E and F). To accommodate the newly inserted CTE-C, residues 853 to 861 of the proximal CTE-N undergo substantial remodeling. Residues 859 to 861 shift downward from the helical bundle and fold into a short β strand, forming a stabilizing β sheet with residues 900 to 902. Reciprocally, residues 853 to 855 move upward to fill the space left by the displacement of the bulky residue W859, which was found to undergo minimal rearrangements in the isolated αE-catenin ABD–F-actin structure, where it restricts rearrangements of CTE-N (Fig. 3, E and F) (*18*, *45*).

### Afadin–αE-catenin interfaces mediating supra-complex binding to F-actin

**Fig. 4.**
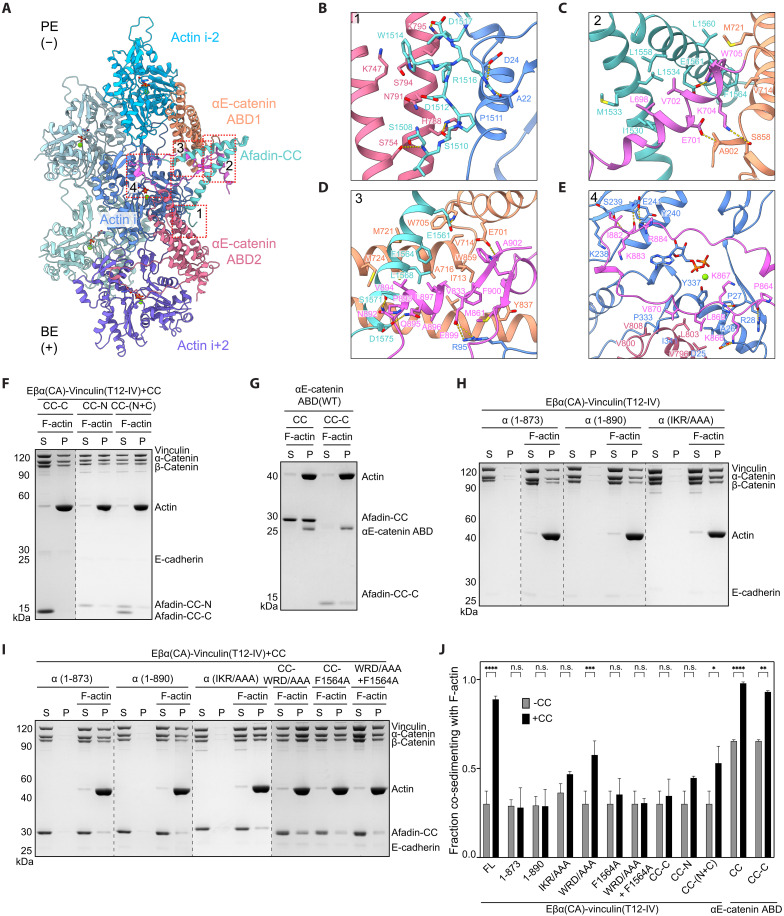
Afadin-CC engagement mediates enhanced binding of the αE-catenin ABD to F-actin. (**A**) Overview of the tripartite interface. BE, barbed end; PE, pointed end. (**B**) Contacts between actin subunit i, αE-catenin ABD2, and afadin-CC’s N-terminal coil. (**C**) Contacts at the interface between the displaced H1 and H2 of αE-catenin ABD1 and afadin-CC. (**D**) Interaction network formed by afadin-CC’s C-terminal coil, ABD1, and actin subunit i. (**E**) Contacts between the CTE of αE-catenin ABD1, actin subunit i, and H4 of αE-catenin ABD2. (**F** and **G**) Co-sedimentation assays analyzing the effects of afadin-CC fragments on F-actin binding by the tetrameric complex (F) and the isolated αE-catenin ABD (G). (**H** and **I**) Co-sedimentation assays analyzing the contributions of specific interfaces to the tetramer’s F-actin binding in the absence (H) and presence (I) of afadin-CC. (**J**) Quantification of (F) to (I). Data are presented as means ± SD of three independent experiments. Conditions were compared by two-way ANOVA with Tukey’s multiple comparison test: n.s., *P* ≥ 0.05; *P < 0.05; **P < 0.01; ***P < 0.001; ****P < 0.0001. Dotted lines indicate stitching interfaces between gels.

Afadin binding and the concomitant structural rearrangements of the αE-catenin ABD establish multiple interfaces likely to strengthen the interaction between the cadherin-catenin complex and F-actin (Fig. 4). At the N terminus of the first afadin coil, a short segment comprising residues 1509 to 1518 forms a tripartite interface with ABD2 and actin subunit i (Fig. 4, A and B). This interface features a combination of hydrogen bonds, van der Waals contacts, and salt bridges. Notably, W1514 from afadin embeds into a groove formed by H3 and H4 of ABD2, while R1516 hydrogen bonds with A22 and D24 of actin i. In addition, D1517 forms a salt bridge with αE-catenin K795.

The rearranged H1 helix of ABD1 is sandwiched by the coiled-coil of afadin and the CTE-N of ABD1. The interaction between H1 and the coiled-coil is mediated by a predominantly hydrophobic interface. Afadin-induced repositioning of H1 places W705 in a newly formed hydrophobic groove between the second coil of afadin and H5 of ABD1. In addition, several hydrophilic H1 residues on the opposite side of the helix make electrostatic interactions with both CTE-N and CTE-C (Fig. 4C).

The folded short α helix of CTE-C (893 to 898) is sandwiched by the C-terminal coil of afadin-CC and H2/H5 of ABD1 (Fig. 4D). The following short β strand (900 to 902) and the resulting β sheet further packs onto the sandwich module, creating an intertwined interaction network primarily driven by hydrophobic interactions. Hydrophobic residues in the CTE-C, including V894, A896, L897, and F900, deeply penetrate into the hydrophobic groove created by H2 and H5, which is further fortified by F1564 and L1568 from afadin, as well as W859 and M861 from CTE-N. In addition to these hydrophobic interactions, the rearranged CTE-C also forms several electrostatic interactions with afadin and F-actin (Fig. 4, C and D).

The conformation of CTE-N residues 862 to 871 remains essentially unaltered after afadin engagement versus the post-bound state (Fig. 3E). The proximal segment electrostatically interacts with actin i, while the distal segment sits in a hydrophobic cleft cradled by actin i and ABD2, where previously reported direct contacts with ABD2 are resolved in the supra-complex structure (Fig. 4E) (*18*, *45*). Beyond this region, a flexible linker enables a patch of previously disordered residues (882 to 884) to align in parallel with a β strand from subdomain 4 of actin i, forming, to our knowledge, a previously unreported interface between αE-catenin and F-actin (Fig. 4, A and E). The contacts feature a salt bridge between αE-catenin R884 and actin E241, backbone hydrogen bonding between αE-catenin K883 and actin S239 and E241, as well as van der Waals contacts involving αE-catenin I882 (Fig. 4E). Collectively, this extensive network of interactions likely directly stabilizes the cadherin-catenin complex’s F-actin binding in the presence of afadin.

### αE-catenin ABD–afadin-CC interface required for AJ actomyosin organization

Although we visualized extensive contacts between residues 1510 to 1602 of the afadin-CC domain (hereafter referred to as afadin-CC-C) and the αE-catenin ABD, residues 1393 to 1510 (afadin-CC-N) were not resolved in our structure. As this sequence is responsible for binding to αE-catenin’s M3 domain (fig. S2D) (*36*), we hypothesized that it could also contribute to activation by promoting ABD release, in a manner conceptually analogous to the vinculin Vh domain–αE-catenin M3 domain interaction. We thus sought to determine the interplay of these two afadin-CC segments, which represent distinct αE-catenin binding sites, in enhancing the cadherin-catenin complex’s F-actin engagement using co-sedimentation assays. Neither isolated afadin-CC-N nor afadin-CC-C alone had a stimulatory effect on F-actin binding by the tetramer [Eβα(CA)-vinculin (T12-IV); Fig. 4, F and J]. This suggests that afadin-CC-C’s direct stabilization of the F-actin binding interface can only occur if αE-catenin ABD release is promoted by afadin-CC-N. In agreement with this model, both afadin-CC and afadin-CC-C potently stimulated F-actin binding by the isolated αE-catenin ABD, which completely lacks autoinhibition (Fig. 4, G and J). Furthermore, a mixture of afadin-CC-N and afadin-CC-C substantially increased F-actin binding by the tetramer, consistent with our proposed hierarchy of activities, albeit less potently than the intact afadin-CC (Fig. 4, F and J). This suggests the physical linkage of these two segments enhances afadin-CC’s overall effect on stimulated F-actin binding, potentially by increasing the local concentration of afadin-CC-C in the vicinity of αE-catenin ABD activated by afadin-CC-N.

We next dissected the afadin-CC-C/αE-catenin ABD interfaces that we visualized by designing mutations to selectively disrupt them in the context of the pentamer featuring IV mutations in vinculin [Eβα(CA)-vinculin (T12-IV)-afadin-CC] and performing co-sedimentation assays. In the refolded αE-catenin CTE-C region, truncation of either the entire CTE-C (874 to 906) or the C-terminal short α helix and β strand (891 to 906) did not affect the F-actin binding of the tetrameric complex [Eβα(CA)–vinculin(T12-IV)] in the absence of afadin-CC, but these lesions completely abolished afadin-CC’s stimulatory effect (Fig. 4, H to J). Moreover, mutations disrupting the interface between the proximal CTE-C (I882A/K883A/R884A, “IKR/AAA”) and F-actin led to a notable reduction in stimulation (Fig. 4, H to J). These results together demonstrate that both the actin and afadin binding regions of αE-catenin’s CTE-C are indispensable for enhancement of F-actin binding by afadin. While CTE-C is highly conserved across metazoans and among different α-catenin isoforms, an equivalent segment is absent in vinculin (fig. S5A). Thus, the CTE-C segment likely plays a conserved role in specifically mediating enhancement of cadherin-catenin complex–F-actin binding through the α-catenin ABD.

In the first coil of afadin-CC, alanine substitutions of the key ABD2 interacting residues W1514, R1516, and D1517 (W1514A/R1516A/D1517A, “WRD/AAA”) substantially decreased F-actin binding stimulation (Fig. 4, I and J). Similarly, a single-alanine substitution of F1564 within the second afadin-CC coil, which is involved in the core of hydrophobic interactions with ABD1, nearly abolished afadin-CC’s stimulatory effect (Fig. 4, I and J). Combining these mutations reduced afadin-CC’s stimulatory effect to an undetectable level (Fig. 4, I and J). These findings suggest that afadin’s interaction with both αE-catenin ABDs is important for its functional modulation of the F-actin binding interface, with ABD1 contacts playing a more pronounced role. Notably, the segments of the afadin coiled-coil observed in our structure are highly conserved among vertebrates, and the key residues involved in αE-catenin interaction are highly conserved from *Drosophila* to humans (fig. S5B), suggesting that it plays a similar role in stabilizing the αE-catenin–F-actin interaction across species. It is worth noting that, while the M3 binding region (CC-N) is also highly conserved in vertebrates, it is absent in *Drosophila*. This indicates that CC-N likely specialized to provide varying regulation of αE-catenin activity in different lineages.

**Fig. 5.**
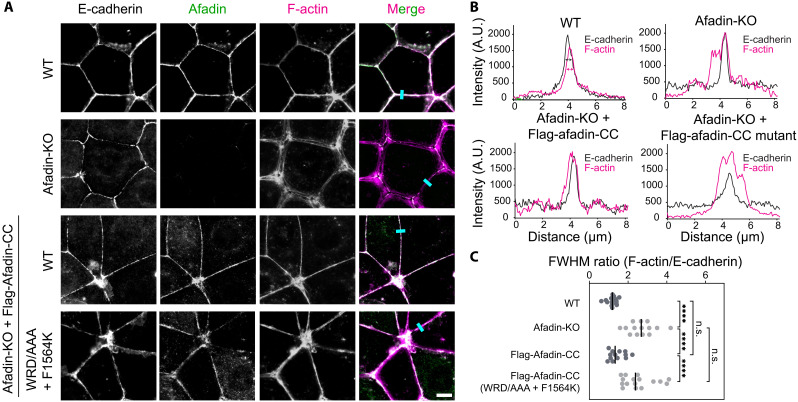
Afadin-CC is required for proper actomyosin organization at AJs. (**A**) Immunofluorescence staining of the indicated proteins at AJs in fixed EpH4 cells. Scale bar, 10 μm. (**B**) Line scan quantification of F-actin and E-cadherin signal along the indicated lines (cyan) in (A). Magenta and green double-headed arrows indicate the full width at half maximum (FWHM) peak intensities for F-actin and E-cadherin, respectively. A.U., arbitrary units. (**C**) Quantification of FWHM ratio of F-actin to E-cadherin. Bars represent means. Data represent three independent experiments (13 ≤ *n* ≤ 15) compared using one-way ANOVA with Fisher’s least significant difference test. n.s., *P* ≥ 0.05; ****P < 0.0001.

In EpH4 mouse mammary epithelial cells, afadin-CC has been shown to be necessary and sufficient for proper actin-myosin (actomyosin) bundle organization at AJs (*29*). In WT cells, afadin co-localizes with E-cadherin at AJs. The actomyosin network, as indicated by F-actin staining, forms a distinct, thick line along the junctions between adjacent cells, implying tight coupling between the cadherin-catenin complex and junctional actomyosin (Fig. 5A and fig. S6A). However, in afadin knockout cells, while E-cadherin maintains its localization to the plasma membrane, the F-actin signal broadens at both bicellular and tricellular junctions, indicating junctional actomyosin disorganization. Previous work has shown that reexpression of afadin-CC alone rescues junctional actomyosin organization (*29*), with this restoration being only partial at tricellular junctions, which sustain higher mechanical loads than bicellular junctions. To quantitatively analyze the magnitude of F-actin signal broadening at bicellular junctions, we performed line scan quantification of the F-actin and E-cadherin signals and then calculated the ratio of the full width at half maximum (FWHM) peak intensities of F-actin to E-cadherin, as previously described (*29*). In WT cells, the ratio was near 1, suggesting the co-localization of F-actin with E-cadherin in an organized band. However, in afadin knockout cells, the ratio was substantially increased, indicating weakened cytoskeleton-AJ coupling (Fig. 5, B and C).

Consistent with a previous study (*29*), we found that reexpressing afadin-CC alone is sufficient to restore the single thick band of F-actin and its co-localization with E-cadherin at bicellular junctions and, to a lesser extent, at tricellular junctions (Fig. 5A and fig. S6). However, expression of mutant afadin-CC deficient in αE-catenin binding (W1514A/R1516A/D1517A/F1564K) failed to restore proper actomyosin architecture at both bicellular and tricellular junctions (Fig. 5 and fig. S6), consistent with this construct’s inability to enhance the cadherin-catenin complex’s binding to F-actin in vitro (Fig. 4I). This suggests that direct interactions between the afadin coiled-coil and αE-catenin’s ABD are necessary for proper cellular actomyosin organizations at AJs.

### Cadherin-catenin–vinculin supra-complex cooperative F-actin binding promoted by afadin

**Fig. 6.**
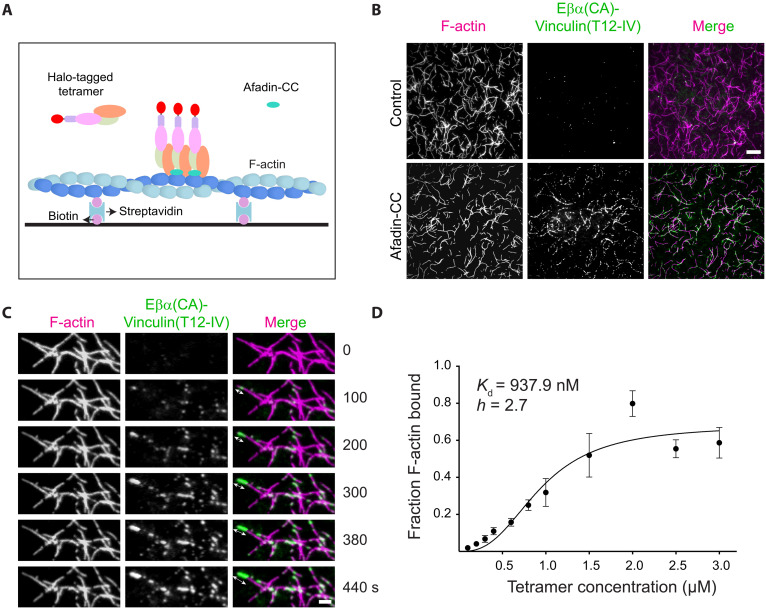
Afadin-CC stimulates cooperative binding of the tetrameric complex to F-actin. (**A**) Schematic of TIRF microscopy assay. (**B**) TIRF snapshots of Atto488-labeled actin (magenta) and Janelia Fluor 549–labeled Eβα(CA)-vinculin(T12-IV) (green) in the absence (top) or presence (bottom) of unlabeled afadin-CC. Scale bar, 5 μm. (**C**) Montage of tetramer patches in the presence of afadin-CC. Double-headed arrows illustrate the bidirectional elongation of a pentamer patch. Scale bar, 2 μm. (**D**) Fraction F-actin bound plotted against concentration of the tetramer in the presence of fourfold excess afadin-CC.

Our finding that afadin-CC bridges adjacent αE-catenin ABDs along F-actin strands led us to hypothesize that it would promote cooperative binding of the tetrameric complex [Eβα(CA)-vinculin (T12-IV)] to F-actin, a behavior previously observed for the isolated αE-catenin ABD but reported to be absent for the trimeric Eβα complex (*9*, *17*). We implemented a TIRF microscopy assay to visualize the tetramer’s F-actin binding dynamics (Fig. 6A). We observed sparsely distributed binding events by TIRF, visible as small puncta of tetramer signal on individual filaments (Fig. 6B). Adding a Halo tag to the N terminus of E-cadherin for fluorescent labeling did not alter afadin-CC’s robust enhancement of the tetramer’s binding to F-actin in co-sedimentation assays (fig. S1F). Correspondingly, addition of afadin-CC also elicited a notable increase in tetramer binding in TIRF assays, which occurred through the formation of discrete patches along filaments (Fig. 6B). Time-lapse imaging revealed bidirectional patch elongation, a hallmark of cooperative filament binding (Fig. 6C). Fitting a saturation binding curve supports cooperative binding [dissociation constant (*K*_d_) = 0.94 μM; Hill coefficient (*h*) = 2.7], similar to that previously reported for the isolated αE-catenin ABD (Fig. 6D) (*9*). These data suggest that afadin-CC potentiates αE-catenin’s intrinsic cooperative F-actin binding in the context of higher-order adhesion assemblies, likely by both relieving autoinhibition through afadin-CC-N and by mediating direct contacts between F-actin bound ABDs through afadin-CC-C.

### Correlation between supra-complex binding along F-actin strands and filament curvature

**Fig. 7.**
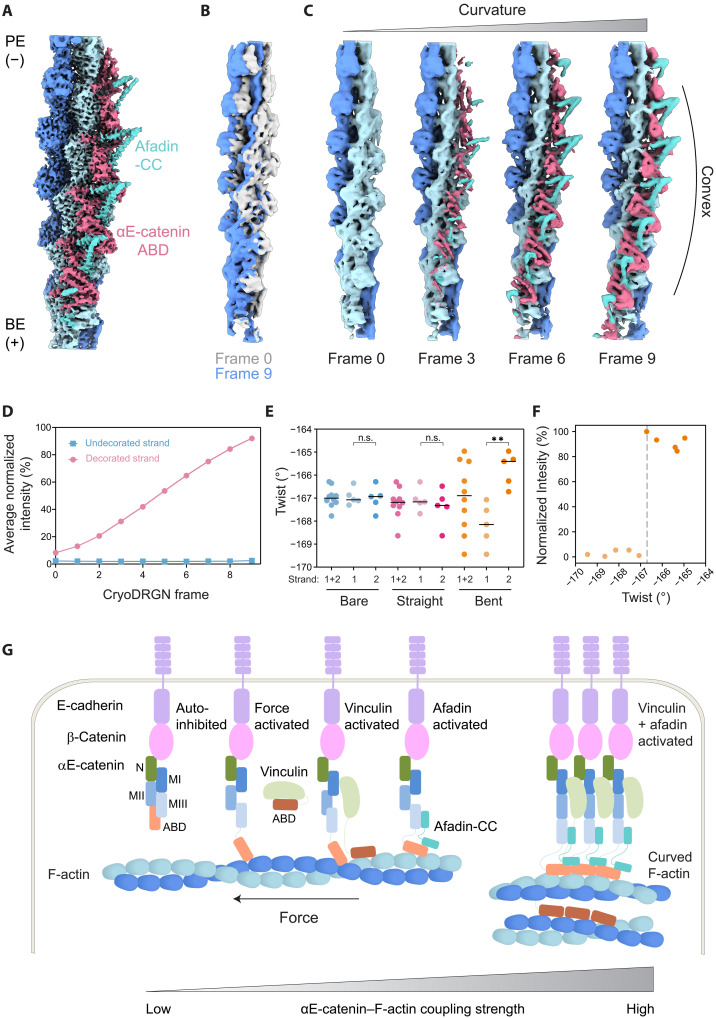
Cooperative binding of the pentameric complex is correlated with F-actin curvature. (**A**) Density map (4.1-Å resolution) that resolves αE-catenin ABDs and bridging afadin-CCs bound along a single F-actin strand. (**B**) Alignment of the first (least curved, shown in gray) and last frame (most curved, shown in cornflower blue) from cryoDRGN variability analysis. Pentamer density was removed to highlight filament curvature. Frames are aligned on the three terminal actin subunits at the barbed end. (**C**) Frames from cryoDRGN variability analysis capture coupling between increasing filament curvature and complex binding. (**D**) Quantification of average complex intensity along each F-actin strand across the cryoDRGN trajectory. (**E**) Quantification of the local twist of the bare filament (“bare”), straight filament with both strands decorated (“straight”), and the most curved filament (frame 9) from cryoDRGN analysis (“curved”). Circles represent the twist measured at each protomer index of the 10 central subunits. Strand 2 corresponds to the decorated strand of the curved filament. Bars represent means. Data were analyzed by one-way ANOVA with Tukey’s multiple comparison test: n.s., *P* ≥ 0.05; **P < 0.01. (**F**) Quantification of complex intensity versus local twist at each protomer index in the curved reconstruction from (D). Vertical dashed line indicates twist of canonical straight F-actin. (**G**) Schematic model of the tunable coupling strength between the cadherin-catenin complex and F-actin, modulated by mechanical force, vinculin, and afadin.

In our previous studies of force-activated F-actin binding by the isolated αE-catenin ABD, we speculated that inter-ABD cooperative contacts could facilitate the protein’s discrimination of mechanical transitions in F-actin (*18*). We recently experimentally confirmed this hypothesis in a study performed contemporaneously with the work presented here (*47*). Our finding that afadin-CC enhances these contacts, as well as our recovery of substantially populated 2D classes featuring curved F-actin decorated along a single strand from our cryo-EM dataset, led us to hypothesize that the full pentamer could biochemically stabilize “architectural remodeling” (*48*), wherein actin subunits are repositioned relative to one another in F-actin’s helical lattice, resulting in overall filament curvature. We therefore conducted detailed structural analysis of the segments contributing to curved F-actin classes, resulting in a 4.1-Å reconstruction (Fig. 7A and table S1). Consistent with 2D analysis, the F-actin in this reconstruction features notable curvature, as well as density for the αE-catenin ABD and afadin-CC solely present along a single strand, occupying the convex side of the curve. Docking the atomic model of the pentamer–F-actin interface derived from the straight reconstruction into this density map did not reveal any notable changes in local binding contacts, suggesting that recognition and stabilization of deformed F-actin likely depends on the discrimination of longer-range filament lattice architectural features by cooperative multi-pentamer assemblies.

To investigate the link between filament curvature and pentamer engagement, we performed variability analysis with cryoDRGN (Materials and Methods). Analysis of a volume series representing the first principal component of variation revealed a clear correlation between increasing curvature and the appearance of pentamer density along the convex strand, suggesting a link between filament deformation and cooperative binding along an individual F-actin strand (Fig. 7, B and C). To quantify this phenomenon, we measured the average normalized intensity of the pentamer density (a proxy for occupancy) along each strand for each frame (Fig. 7D). To analyze architectural remodeling of the filament lattice accompanying this deformation, we also measured the instantaneous helical parameters (twist, the rotation angle between neighboring subunits along the helical lattice, and rise, the distance between the centroids of neighboring subunits projected along the filament axis) at each subunit index in the cryoDRGN frame featuring maximal curvature (frame 9). As points of comparison, from the same dataset, we also asymmetrically reconstructed a bare filament region and a fully decorated straight filament region spanning an equivalent number of subunits (fig. S7, A and B, and table S1).

Previous cryo-EM work from our lab (*49*), as well as earlier theoretical studies (*50*), showed that uniplanar curvature in F-actin is associated with asymmetric twist values between the promotors of the two strands composing the filament, a phenomenon known as “twist-bend coupling.” In a study contemporary with the work performed here, we found that myosin motor forces additionally elicit asymmetric changes in rise, producing a superhelical spiral form of F-actin (*47*). As anticipated, twist is highly uniform for both the bare filament and the fully decorated straight filament. In contrast, although the overall average twist of the maximal curvature frame is similar to the straight filaments, the average twist of each strand is distinct in the maximal curvature volume, consistent with prior reports of twist-bend coupling in F-actin (*49*, *50*). The highly decorated strand is substantially under-twisted (Fig. 7E), leading to a correlation between subunits featuring a lower twist magnitude and high pentamer intensity (Fig. 7F). Collectively, this suggests cooperative pentamer binding either induces or stabilizes filament curvature, with preferential binding occurring along the convex face of curved filament regions.

In our contemporary study of the impacts of myosin-generated forces on F-actin structure, we found that the isolated αE-catenin ABD preferentially engages positions featuring extended rise (*47*). To assess whether the pentameric complex induces or stabilizes similar lattice rearrangements, we analyzed the correlation between instantaneous helical rise and pentamer intensity. While the bare filament displayed uniform helical rise, both the fully decorated straight filament and the maximal curvature filament displayed increased rise along one strand and decreased rise along the other (fig. S7C), nevertheless maintaining an overall average similar to bare F-actin (*49*). In the curved filament, the subunits featuring increased rise are correlated with high pentamer intensity (fig. S7D), consistent with the behavior of the isolated ABD in the presence of myosin forces (*47*). However, in the fully straight filament, all subunits are equivalently decorated, suggesting that rise changes are not the sole determinant of asymmetric engagement by the pentamer. One potential explanation for this difference between the pentameric complex and the isolated αE-catenin ABD is that the fully decorated reconstruction represents a mixture of different species featuring variable pentamer occupancy, which we were unable to sort via current 3D classification or variability methods. Nevertheless, these data are broadly consistent with afadin-CC stabilizing cooperative, asymmetric binding by the cadherin-catenin complex to curved F-actin, reminiscent of force-activated binding by the αE-catenin ABD.

## DISCUSSION

This study provides biochemical and structural insights into how the intrinsic actin-binding activity of the core cadherin-catenin complex can be modulated by additional adhesion factors, revealing mechanisms that could tune AJ-cytoskeleton coupling strength for function across varying mechanical conditions (Fig. 7G). Our biochemical data are broadly consistent with a model in which the association of vinculin and afadin-CC cumulatively relieve autoinhibition of αE-catenin’s ABD, with the convergence of both proteins supporting substantially higher F-actin engagement than either factor alone. We further found evidence of supra-complex composition-dependent interplay between the vinculin ABD and the αE-catenin ABD. The vinculin ABD serves as the dominant actin-binding moiety in the tetrameric cadherin-catenin–vinculin complex, consistent with a recent study (*42*), while the αE-catenin ABD plays a predominant role in the pentameric cadherin-catenin–vinculin–afadin complex. This suggests that the vinculin and αE-catenin ABDs may have dynamically varying functions as AJs undergo compositional and mechanical remodeling in vivo, an important topic for future mechanistic cell biology studies, which can be guided by the biophysical framework introduced here.

Our structural studies show direct modulation of the αE-catenin–F-actin interface by the afadin-CC, providing an additional mechanism for stabilizing the cadherin-catenin complex’s F-actin binding. Afadin-CC engages flexible αE-catenin ABD elements implicated in force-based enhancement of αE-catenin’s F-actin binding (*18*), supporting a model in which afadin biochemically co-opts α-catenin’s mechanical regulatory mechanisms to achieve constitutively strong, stable binding. Interpreted through the framework where H1 undocking from the αE-catenin ABD helical bundle mediates catch bonding, while CTE dynamics mediate force-activated binding, our data predict that force is unlikely to additionally stabilize F-actin binding once the pentameric complex featuring afadin-CC has fully engaged. Nevertheless, by pre-organizing these structural elements in the αE-catenin ABD, force is likely to promote afadin association with the complex. Functionally, this could facilitate persistent maximal AJ-cytoskeletal coupling at cell-cell junctions bearing consistently high mechanical loads. Selective disruption of specific afadin-CC–αE-catenin ABD contacts that we have identified, which are highly conserved, can facilitate precise dissection of the functions of this interface in tissue dynamics across species.

Additionally, we find that afadin-CC mediates contacts between neighboring αE-catenin ABDs, promoting the formation of cooperative assemblies along individual F-actin strands in filament segments featuring nanoscale curvature. While, to our knowledge, this binding mode has not previously been described, other actin-associated proteins, including IQGAP and septins, have been reported to promote F-actin bending, albeit at the micron rather than nanometer scale (*51*, *52*). Structural studies of these factors in complex with F-actin will be required to decipher whether they use similar mechanisms. While we speculate that the pentamer’s recognition/stabilization of F-actin curvature represents further cooption of mechanical regulation by afadin, it could also feasibly serve additional functions. In cells, the complex is localized to the plasma membrane, where individual strands of actin filaments adjacent to the membrane are locally accessible (*16*, *29*, *53*). Forming assemblies along F-actin strands thus represents a cooperativity mechanism compatible with physiological geometric restrictions. Moreover, afadin is frequently localized to sites featuring high local membrane curvature, such as tricellular junctions (*30*, *54*), where stabilizing F-actin curvature could conceivably enhance AJ-cytoskeleton linkages by aligning the contour of filaments with that of the membrane. Nanoscale imaging studies, such as cryo–electron tomography of AJs with varying architectures and afadin abundance, will be necessary to dissect the detailed functional links between local F-actin morphology and AJ-cytoskeleton coupling.

## MATERIALS AND METHODS

### Cell culture

WT and afadin knockout EpH4 cell lines were a gift from Y. Takai at Kobe University, Japan. Human embryonic kidney (HEK) 293T cells were generously provided by Kun-Liang Guan at the University of California, San Diego. The cells were maintained in Dulbecco’s modified Eagle’s medium (Thermo Fisher Scientific, 11995-065) supplemented with 10% heat-inactivated fetal bovine serum (Sigma-Aldrich, F4135) and 1× antibiotic-antimycotic (Thermo Fisher Scientific, 15240-062). FreeStyle 293-F cells (Thermo Fisher Scientific, R79007) were cultured in FreeStyle 293 expression medium (Thermo Fisher Scientific, 12338018).

### Plasmids and cloning

The cDNAs of mouse E-cadherin (NM_004360.5; Addgene, no. 18804; gift of B. Weinberg), β-catenin (NM_007614.3; Addgene, no. 20140; gift of V. Vasioukhin), and αE-catenin (NM_009818.1; Addgene, no. 20139; gift of V. Vasioukhin) were obtained from Addgene. The cDNA of mouse afadin (NM_010806.1) was purchased from Horizon Discovery (MMM1013-202707312). The cDNA of human vinculin (NM_003373.4) was polymerase chain reaction amplified from a cDNA library prepared from HEK293T cells. Full-length coding sequences or fragments of E-cadherin, β-catenin, αE-catenin, vinculin, and afadin (table S2) were individually cloned into a modified pCAG mammalian expression vector, either untagged or tagged with an N-terminal green fluorescent protein (GFP) or Flag tag (DYKDDDDK) followed by a tobacco etch virus (TEV) protease cleavage site (*55*). To express GFP and Halo-tagged E-cadherin, a Halo tag was inserted immediately after the TEV cleavage site. For lentiviral production, the cDNAs of afadin constructs with an N-terminal Flag tag were cloned into a doxycycline-inducible pCDH-EF1a-MCS vector carrying a puromycin selection marker. The reverse tetracycline-responsive transcriptional activator (rtTA) was expressed in a separate pCDH vector with G418 selection. Both vectors were generously provided by Kun-Liang Guan at the University of California, San Diego. All WT and mutant clones were generated by Gibson assembly (New England Biolabs, E2611) and validated by DNA sequencing.

### Protein expression and purification

FreeStyle 293-F cells were maintained in FreeStyle 293 expression medium and transfected at a density of 1.8 × 10^6^ cells/ml. For 1 liter of cells, 1 mg of plasmid encoding the protein of interest was mixed with 3 ml of PEI MAX (Polysciences, 24765-1) in 40 ml of fresh expression medium and incubated for 20 min at room temperature before transfection. Cells were harvested 72 hours after transfection. The cadherin-catenin complex was co-expressed by co-transfecting plasmids featuring GFP-tagged E-cadherin, untagged β-catenin, and untagged αE-catenin at a mass ratio of 1:2:2. The cadherin-catenin–vinculin complex was co-expressed by co-transfecting plasmids of Flag-tagged vinculin, GFP or GFP and Halo-tagged E-cadherin, untagged β-catenin, and untagged αE-catenin at a mass ratio of 1:2:4:4. Afadin constructs were expressed individually, and full pentameric complexes were reconstituted by mixing cadherin-catenin complexes with afadin fragments. Vinculin constructs were also expressed and purified alone for comparative binding assays.

Cells were lysed in lysis buffer [50 mM tris-HCl (pH 8.0), 150 mM NaCl, 2 mM MgCl_2_, 0.2% CHAPS, 2 mM β-mercaptoethanol, 5 mM adenosine 5′-triphosphate (ATP), 1 mM phenylmethylsulfonyl fluoride, aprotinin (1 μg/ml), leupeptin, and pepstatin]. β-Mercaptoethanol was excluded from lysis buffer for Flag affinity purification. Cell debris was removed by centrifugation at 20,000*g* for 30 min at 4°C. GFP-tagged cadherin-catenin complexes, afadin constructs, and vinculin were affinity purified from supernatants using an anti-GFP nanobody coupled to NHS-activated Sepharose 4 Fast Flow resin (anti-GFP beads) (*56*). The cadherin-catenin–vinculin complex was first affinity purified from supernatant using Flag M2 beads (Sigma-Aldrich, A2220). The bound complex was eluted with Flag peptide (200 μg/ml; Sigma-Aldrich, F3290) and then further affinity purified using anti-GFP beads. The tags for all affinity-purified proteins were removed by TEV (0.1 mg/ml) protease digestion (*57*), and then the cleaved proteins were eluted using elution buffer [25 mM tris-HCl (pH 8.0), 150 mM NaCl, and 0.1% CHAPS]. Afadin, vinculin, and the cadherin-catenin complex were further purified by anion exchange chromatography on a MonoQ 5/50 GL column (GE Healthcare), followed by size exclusion chromatography on a Superdex 200 increase 10/300 GL column (GE Healthcare). The cadherin-catenin–vinculin complex was directly polished by size exclusion chromatography on a Superose 6 10/300 column (GE Healthcare) without initial purification by anion exchange. Size exclusion columns were equilibrated with size exclusion buffer containing 10 mM tris-HCl (pH 8.0), 100 mM NaCl, and 3 mM dithiothreitol (DTT).

Chicken skeletal muscle actin was prepared as previously described (*58*) and stored at 4°C in G-Ca buffer: 2 mM tris-HCl (pH 8.0), 0.5 mM DTT, 0.1 mM CaCl_2_, 0.2 mM ATP, and 0.01% NaN_3_. F-actin was polymerized by mixing monomeric actin [diluted in G buffer: 2 mM tris-HCl (pH 8.0), 0.5 mM DTT, 0.2 mM ATP, and 0.1 mM MgCl_2_] with KMEI buffer [50 mM KCl, 1 mM MgCl_2_, 1 mM EGTA, and 10 mM imidazole (pH 7.0)] and incubated at room temperature for 1 hour.

### F-actin co-sedimentation assays

The cadherin-catenin complexes (4 μM), either alone or mixed with 6 μM afadin fragment, were diluted in KMEI buffer and precleared by ultracentrifugation at 80,000 rpm (278,000*g*) in a TLA-100 rotor (Beckman Coulter) for 15 min at 4°C. The supernatant (50 μl) was mixed with an equal volume of 10 μM F-actin. The mixture was incubated for 30 min at room temperature and then ultracentrifuged at 80,000 rpm (278,000*g*) for 30 min at 4°C. The supernatant (40 μl) was mixed with 10 μl of 5× SDS–polyacrylamide gel electrophoresis (PAGE) sample buffer [50 mM tris-HCl (pH 8.0), 50 mM NaCl, 2% SDS, 0.025% bromophenol blue, 10% glycerol, and 5% β-mercaptoethanol]. The pellet was washed twice with F-actin buffer (KMEI and 0.5 mM DTT) and dissolved in 125 μl of 1× SDS-PAGE sample buffer. Samples were separated by SDS-PAGE, stained with Coomassie brilliant blue, scanned on a LI-COR Odyssey scanner (LI-COR), and quantified with Fiji (*59*).

### Pull-down assays

All proteins were expressed in FreeStyle 293-F cells. Flag-tagged afadin fragments were first bound to Flag M2 beads by affinity purification as described above. Cells expressing GFP-tagged individual domains of αE-catenin or the cadherin-catenin-vinculin complex were lysed in lysis buffer and spun at 20,000*g* for 30 min at 4°C. The supernatants were collected and then incubated with afadin-bound Flag M2 beads. After 30 min of incubation, the beads were washed three times with lysis buffer, dissolved in 50 μl of 1× SDS-PAGE sample buffer and analyzed by SDS-PAGE as described above.

### Stable cell line generation

Lentiviruses were produced in HEK293T cells by co-transfecting the pCDH lentiviral vector with the packaging plasmids PsPAX2 and pMG2.g at a mass ratio of 4:3:1 using lipofectamine 3000 (Thermo Fisher Scientific, L3000008). Forty-eight hours after transfection, the culture medium containing the virus was harvested and filtered through a 0.45-μm–pore-size filter. Polybrene (5 μg/ml; Sigma-Aldrich, TR-1003-G) was added to enhance infection efficiency. Afadin knockout EpH4 cells were infected with an equal amount of lentiviruses encoding Flag-tagged afadin constructs and rtTA. Forty-eight hours after infection, cells were selected with puromycin (2 μg/ml; Thermo Fisher Scientific, A1113803) and G-418 (800 μg/ml; Sigma-Aldrich, 108321-42-2) for 7 days. Surviving cells were diluted for single-clone selection. Afadin expression was induced by addition of doxycycline (0.05 μg/ml; Sigma-Aldrich, D9891) to single-cell derived clones. Cells were analyzed by Western blot or immunostaining 24 hours after induction.

### Immunofluorescence imaging

EpH4 cells at 90% confluency were fixed with 4% formaldehyde (Thermo Fisher Scientific, 28908) in cytoskeleton buffer [CB; 100 mM MES (pH 6.1), 1.38 M KCl, 30 mM MgCl_2_, and 20 mM EGTA] for 15 min, permeabilized in 0.1% Triton X-100 in CB for 10 min, and then incubated with 100 mM glycine in CB for 10 min. After washing with Dulbecco’s phosphate buffered saline (DPBS, Gibco, 14190-144), cells were blocked in 2% bovine serum albumin (BSA; Gemini Bio-Products, 700-101P) in DPBS for 1 hour at room temperature. Blocked cells were incubated with primary antibodies in DPBS and 2% BSA overnight at 4°C. After washing with DPBS, cells were incubated with secondary antibodies at room temperature for 1 hour and washed with DPBS three times. The following antibodies were used for immunostaining: mouse anti-FLAG monoclonal antibody (mAb; 1:200; Sigma-Aldrich, F3165); rat anti–E-cadherin mAb (1:500, Thermo Fisher Scientific, 13-1900); mouse anti-afadin mAb (1:200; Santa Cruz Biotechnology, sc-74433); Alexa Fluor 488 goat anti-mouse (1:250; Thermo Fisher Scientific, A11029); Alexa Fluor 488 goat anti-rabbit (1:250; Thermo Fisher Scientific, A32731); and Alexa Fluor 647 donkey anti-rat (1:250; Jackson ImmunoResearch, 712-605-153). Alexa Fluor 568 Phalloidin (1:40; Thermo Fisher Scientific, A12380) was used to visualize F-actin. Image series with a depth range of 1.2 μm and step size of 0.2 μm along the *z* axis were acquired on a Nikon Ti-E microscope equipped with a CFI Apo 100× oil immersion objective (numerical aperture, 1.49). Epifluorescence illumination was provided by a light-emitting diode illuminator (Lumencor). Images were acquired on a Zyla 4.2 sCMOS camera (Andor) and quantified using Fiji (*59*).

### Western blotting

Western blots were performed following standard protocols. EpH4 cells were washed with DPBS once and lysed in 1× SDS-PAGE sample buffer. Samples were separated by SDS-PAGE and transferred onto a nitrocellulose membrane (Bio-Rad, 1620115). The membranes were blocked with SuperBlock blocking buffer (Thermo Fisher Scientific, 37537) and subsequently incubated with primary antibodies and secondary antibodies, both diluted in SuperBlock T20 blocking buffer (Thermo Fisher Scientific, 37536). The membranes were scanned on a LI-COR instrument. The following antibodies were used: mouse anti-FLAG mAb (1:1000; Sigma-Aldrich, F3165); rabbit anti-afadin mAb (1:1000; Thermo Fisher Scientific, 700193); mouse anti–glyceraldehyde-3-phosphate dehydrogenase mAb (1:2000; Abcam, ab8245); IRDye 800CW goat anti-mouse (1:15,000; LI-COR, 926-32210); and IRDye 800CW goat anti-rabbit (1:15,000; LI-COR, 926-32211).

### TIRF microscopy clustering assay and image analysis

No. 1.5 24 mm–by–60 mm glass coverslips were cleaned by sequential sonication in 100% acetone for 30 min, 100% ethanol for 10 min, and 2% Hellmanex for 120 min. After rinsing with MilliQ water, the coverslips were functionalized by incubating with poly(ethylene glycol) monomethyl ether (mPEG)-silane (0.9 mg/ml; Laysan Bio, MPEG-SIL-5000), biotinated mPEG-silane (0.1 mg/ml; Laysan Bio, Biotin-PEG-SIL-5 K), 10 mM HCl, and 96% ethanol overnight. Subsequently, the coverslips were rinsed with 100% ethanol and MilliQ water, air dried, and stored in a container filled with nitrogen gas.

F-actin was prepared by copolymerizing 0.72 μM Atto488-labeled and 0.08 μM biotin-labeled monomeric actin (Hypermol, 8153-02 and 8109-01, both diluted in G buffer) at room temperature for 60 min. The Halo-tagged tetrameric Eβα(CA)–vinculin(T12-IV) complex was fluorescently labeled with Janelia Fluor 549 HaloTag ligand (Promega, GA1110) at a molar ratio of 1:2 at room temperature for 4 hours. After removing free dye using a Pierce dye removal column (Thermo Fisher Scientific, 22858), the labeled complex was diluted to 4 μM in imaging buffer, consisting of motility buffer [MB; 20 mM Mops (pH 7.4), 5 mM MgCl_2_, 0.1 mM EGTA, 50 mM NaCl, and 1 mM DTT], supplemented with 15 mM glucose, glucose oxidase (100 μg/ml), and catalase (20 μg/ml). Before imaging, the labeled complex was precleared by ultracentrifugation at 80,000 rpm (278,000*g*) for 12 min at 4°C in a TLA100 rotor.

Imaging wells were assembled by affixing a CultureWell gasket (GraceBio, 103280, 1 mm in thickness and 6 mm in diameter) onto the cover slip surface. After blocking with casein (1 mg/ml; Sigma-Aldrich, C0406) in MB for 1 hour, the well was incubated with 1 μM streptavidin (Sigma-Aldrich, 189730) in MB for 2 min, rinsed with MB, coated with labeled F-actin for 1 min, and rinsed again with MB. The labeled tetramer, either alone or with dark afadin-CC at four times the tetramer’s concentration, was introduced to replace MB. The well was either immediately mounted for time-lapse imaging or placed in a humidified container for 10 min before individual snapshot imaging.

Dual-color TIRF imaging was conducted using a Nikon Ti-E microscope equipped with an H-TIRF motorized module and NIS-Elements software (Nikon). F-actin and the Halo-tagged tetrameric complex were excited by 488 and 561-nm lasers (Agilent), respectively. For time-lapse imaging, frames were acquired every 2 s using a CFI Apo 100X TIRF oil immersion objective (numerical aperture, 1.49), a quad filter (Chroma), and an iXon EMCCD camera (Andor). Individual snap shots were acquired with identical exposure settings.

Image analysis was performed with custom Python scripts using functions from the scikit-image package (*60*). Uneven illumination in the tetramer channel was corrected using rolling-ball background subtraction with a ball radius of 100 pixels. F-actin and tetramer masks were generated by thresholding (Yen method) and binarizing the corresponding channels. A mask corresponding to tetramer specifically bound to F-actin (“bound tetramer mask”) was then obtained through the logical conjunction of the actin and tetramer masks. The tetramer bound fraction of F-actin was calculated by dividing the area of the bound tetramer mask by the area of the total F-actin mask. The fraction bound was then plotted versus tetramer concentration and fit by the Hill equation (*9*).

### Cryo-EM sample preparation

The tetrameric Eβα(CA)–vinculin(T12-IV) complex (2.5 μM) and afadin-CC (5 μM) were premixed before use. Three microliters of 0.6 μM F-actin [polymerized in the presence of 0.01% NP-40 substitute (Roche) to reduce ice thickness] was applied onto a freshly plasma cleaned C-flat 1.2/1.3 holey carbon Au 300 mesh grid (Electron Microscopy Sciences) in a Leica EM GP plunge freezing apparatus operating at 25°C and 100% humidity. After 1 min of incubation, 3 μl of the premixed protein complex was added and mixed thoroughly with the F-actin drop. After an additional 2 min of incubation, the grid was blotted from the back with Whatman no. 5 filter paper for 5 s and plunge frozen in liquid ethane.

### Cryo-EM and cryo-ET data acquisition

Cryo-EM data were acquired on a Thermo Fisher Titan Krios transmission electron microscope operating at 300 kV equipped with a Gatan K2-summit detector using SerialEM (*61*). All image stacks were recorded at a nominal magnification of ×29,000 in super-resolution mode with a calibrated pixel size of 1.03 Å at the specimen level (super-resolution pixel size of 0.515 Å/pixel). Each exposure was dose fractionated across 40 frames with a total electron dose of 61 *e*^−^/Å^2^ (1.53 *e*^−^/Å^2^ per frame) and a total exposure time of 10 s. The target defocus values ranged from −0.8 to −2.0 μm. Exposures were collected using the beam tilt/image shift strategy, targeting nine holes per stage translation.

Cryo-ET data were obtained on a spherical-aberration (Cs)–corrected Titan Krios transmission electron microscope operating at 300 kV equipped with a Gatan K3 direct electron detector and a BioQuantum energy filter (slit width 20 eV). The tilt series was recorded using a dose-symmetric scheme (*62*) from −60° to 60° with a tilt increment of 3°. Exposures at each tilt angle were acquired at a nominal magnification of ×26,000, corresponding to a calibrated pixel size of 2.6 Å at the specimen level (super-resolution pixel size of 1.3 Å/pixel). Each exposure was fractionated into 12 frames (0.22 *e*^−^/Å^2^ per frame) across an exposure time of 0.6 s, with a total electron dose across the tilt series of 107.82 *e*^−^/Å^2^. The tilt series was collected at a target defocus value of −3.5 μm.

### Cryo-EM image processing

A total of 11,061 movie stacks were motion corrected with a binning factor of 2 (resulting in a 1.03-Å pixel size) using MotionCor2 (*63*). Contrast transfer function (CTF) estimation was carried out with CTFFIND4 (*64*). The signal from bound cadherin-catenin-vinculin complexes prevented standard filament pickers from accurately tracing the filament center. Therefore, particle picking was conducted using a customized neural network–based approach, adapting our previously established approach for processing actin filaments (*49*). Specifically, a library of 312 synthetic volumes was created featuring actin filaments that were either bent or straight. These filaments had varying decoration patterns of a coarse cadherin-catenin complex model that we constructed by aligning crystal structures of individual components [Protein Data Bank (PDB) IDs: 1I7X, 1DOW, and 4IGG] (*44*, *65*, *66*). To encompass plausible cadherin-catenin complex decoration patterns, three potential orientations of the complex relative to F-actin were generated, and, for each complex orientation and F-actin curvature, a set of filaments was generated with one bare filament, one fully decorated filament, and partially decorated filaments with random occupancies. This library was used to train a neural network that successfully segmented F-actin at the exclusion of neighboring signal from the flexibly bound cadherin-catenin complex (fig. S3). Filament splines were traced through these segmented micrographs to generate initial picks.

A total of 1,524,620 picked coordinates were imported into RELION-3.1 (*67*) and extracted with a box size of 448 pixels and then binned by 2. The extracted particles were imported into cryoSPARC v4.2 (*68*) for reference-free 2D classification. A total of 521,858 particles from high-quality 2D classes were selected for ab initio reconstruction and masked homogeneous refinement. The refined particles were imported back into RELION-3.1 and then symmetry expanded with a helical twist of −167°, rise of 27 Å, and 23 asymmetric actin subunits. This effectively captured nearly all the filaments in the dataset. After duplicate removal, 6,729,146 particles were re-extracted with a box size of 448 pixels, binned by 4, and subjected to reference-free 2D classification. High-quality 2D classes displaying additional densities along both strands of the filament were selected and subjected to unsupervised 3D classification while applying helical symmetry. Particles with clear filament decoration were refined and symmetry-expanded by applying a helical twist of −167° and a rise of 27 Å across three asymmetric actin subunits. After duplicate removal, particles underwent multiple rounds of masked 3D classification without image alignment. Particles exhibiting clear afadin-CC density (470,416 particles) were re-centered on residue K889 of the αE-catenin ABD, re-extracted with a box size of 448 pixels without binning, and subjected to CTF refinement, Bayesian polishing, and masked 3D auto-refinement, resulting in a postprocessed density map at 2.9-Å resolution. To enhance the resolution of the αE-catenin CTE, particles underwent another round of focused 3D classification with a tight mask covering only the densities of the CTE and afadin-CC. One high-quality class with 99,745 particles was selected and refined to a resolution of 3.1 Å.

2D classes displaying additional densities along one strand of the filament were re-extracted with a box size of 768 pixels and then binned by 4. Following unsupervised 3D classification, particles showing the best occupancy of additional densities were re-extracted with a box size of 448 pixels and subjected to masked 3D auto-refinement by applying helical symmetry. The refined particles were symmetry expanded by applying a helical twist of 26° and rise of 55 Å across three actin subunits. After duplicate removal, particles were re-extracted with a box size of 512 pixels and subjected to masked 3D auto-refinement without applying helical symmetry, resulting in a density map at 4.4-Å resolution. After one round of supervised 3D classification, the single class with high afadin-CC occupancy was selected (44,548 particles) for subsequent CTF refinement, Bayesian polishing and masked 3D auto-refinement, yielding a postprocessed density map at 4.1-Å resolution. The refined particles were used for continuous conformational variability analysis using cryoDRGN (*69*).

### Cryo-ET reconstruction

Individual frame motion correction and CTF estimation of summed tilt exposures were performed in Warp (*70*). The tomogram was reconstructed using the IMOD software package (*71*). The tilt series was binned by 3 to a final pixel size of 7.8 Å and aligned using the patch-tracking approach. The tomogram was reconstructed using a SIRT-like filter, equivalent to eight iterations.

### Model building and refinement

The models of αE-catenin ABD bound F-actin (PDB: 6UPV) and the coiled-coil of afadin predicted by AlphaFold (*46*) were rigid body docked into the 3.1-Å-resolution density map using UCSF Chimera (*72*). Subsequently, the complete model was manually built and adjusted in Coot, flexibly fitted with ISOLDE in UCSF ChimeraX (*73*, *74*), refined using Phenix.Real_space_refine, and validated with MolProbity as implemented in Phenix (*75*, *76*).

### Local helical parameter measurements

Filament twist and rise were measured using a custom Python script described previously (*49*). In brief, a model containing 15 actin subunits was built by rigid-body fitting each individual actin subunit (PDB: 7R8V, chain B) into the density map of each analyzed filament reconstruction in Chimera. Three copies of each model were iteratively aligned on the four terminal actin subunits at the pointed end. After deleting the overlapping subunits from the two end copies, an extended filament model with 37 subunits was created and used for twist and rise measurement. To avoid edge effects, only the twist and rise values of the central 10 subunits were retained for further analysis.

### Occupancy measurements

Atomic models of one actin subunit with bound αE-catenin–ABD and afadin-CC were individually fit into each reconstructed frame of the first principal component of the cryoDRGN variability trajectory. Fitting for sites with low or no αE-catenin–ABD and afadin-CC density was driven by the actin density. Density for the filament and the bound complex was separated using the “split map” command in Chimera. The integrated intensity of each segmented bound complex sub-volume was computed and then normalized to the site with the highest integrated intensity. As the bound αE-catenin–ABD and afadin-CC at this site had approximately the same intensity as F-actin, this measurement serves as a proxy for occupancy. The mean-normalized intensity for each strand of each reconstructed frame from the cryoDRGN trajectory was then computed.

### Graphics

Figures and movies were generated with UCSF ChimeraX (*74*) and Fiji. Multiple sequence alignments were performed with Clustal Omega (*77*) and rendered with Jalview (*78*).

### Statistical analysis

All statistical analysis and plotting were performed in GraphPad Prism 10 (La Jolla, CA, USA). Details of statistical tests are presented in the figure legends. *P* values less than 0.05 were considered significant.
