# Supplementary material for: Afadin mediates cadherin-catenin complex clustering on F-actin linked to cooperative binding and filament curvature
Source: Sci Adv. 2025 Feb 14;11(7):eadu0989. doi: 10.1126/sciadv.adu0989 (PMC11827635; doi:10.1126/sciadv.adu0989)
Supplement: Supplementary file 1 — Figs. S1 to S7 Tables S1 and S2 Legends for movies S1 to S3 [file sciadv.adu0989_sm.pdf]

Science Advances
AAAS

Supplementary Materials for

Afadin mediates cadherin-catenin complex clustering on F-actin linked to cooperative binding and filament curvature

Rui Gong *et al.*

Corresponding author: Rui Gong, rgong@rockefeller.edu; Gregory M. Alushin, galushin@rockefeller.edu

Sci. Adv. **11**, eadu0989 (2025)
DOI: 10.1126/sciadv.adu0989

The PDF file includes:

Figs. S1 to S7
Tables S1 and S2
Legends for movies S1 to S3

Other Supplementary Material for this manuscript includes the following:

Movies S1 to S3

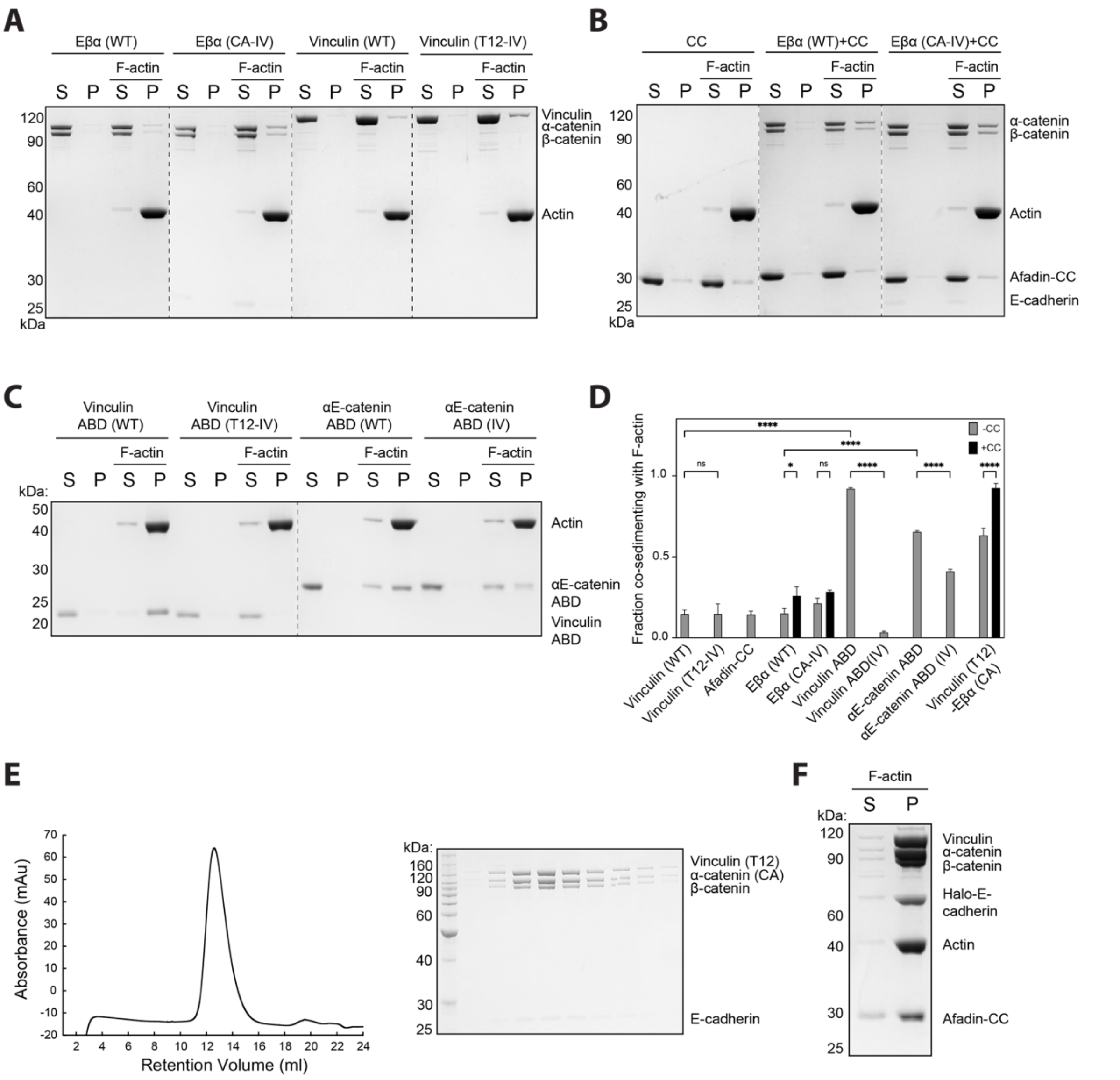

Fig. S1. F-actin binding activities of the cadherin-catenin complex, vinculin and afadin-CC.

(**A**) Co-sedimentation assays of indicated proteins and protein complexes with F-actin. Vinculin(WT), wild-type vinculin; α(WT), wild-type αE-catenin. All other abbreviations are the same as in Figure 1. Dotted lines indicate stitching interfaces between gels. (**B**) Quantification of A. Data are presented as mean ± SD of three independent experiments, compared via two-way ANOVA with Tukey's multiple comparison test. NS, $p \geq 0.05$; $^*p < 0.05$; $^{****}p < 0.0001$. (**C**) Left: Size exclusion chromatography of the tetrameric vinculin(T12)-Eβα(CA) complex. Right: analysis of peak fractions by SDS-PAGE. (**D**) Co-sedimentation of Halo-tagged Eβα(CA)-vinculin(T12-IV) with F-actin in the presence of afadin-CC.

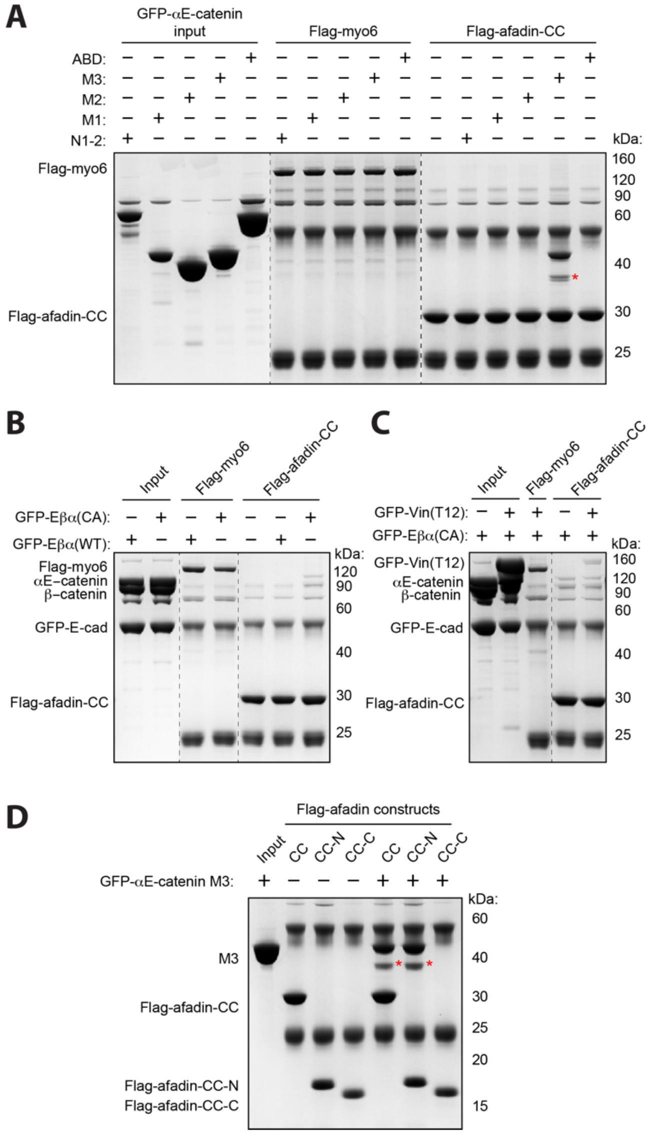

Fig. S2. Afadin-CC forms a pentameric complex with vinculin (T12)-Eβα(CA) through its direct interaction with the M3 domain of αE-catenin.

(**A**) Pull-down assay demonstrates specific binding between afadin-CC and the M3 domain of αE-catenin. The Flag-tagged motor domain of myosin-6 (Flag-myo6) was used as a negative control. The red asterisk indicates nonspecific binding. (**B**) Pull-down assay showing binding between Flag-tagged afadin-CC and GFP-tagged Eβα(CA). (**C**) Pull-down assay showing the interaction between afadin-CC and Eβα(CA)-vinculin(T12). (**D**) Pull-down assay showing the association between afadin-CC-N and the M3 domain of αE-catenin. Red asterisks indicate nonspecific binding.

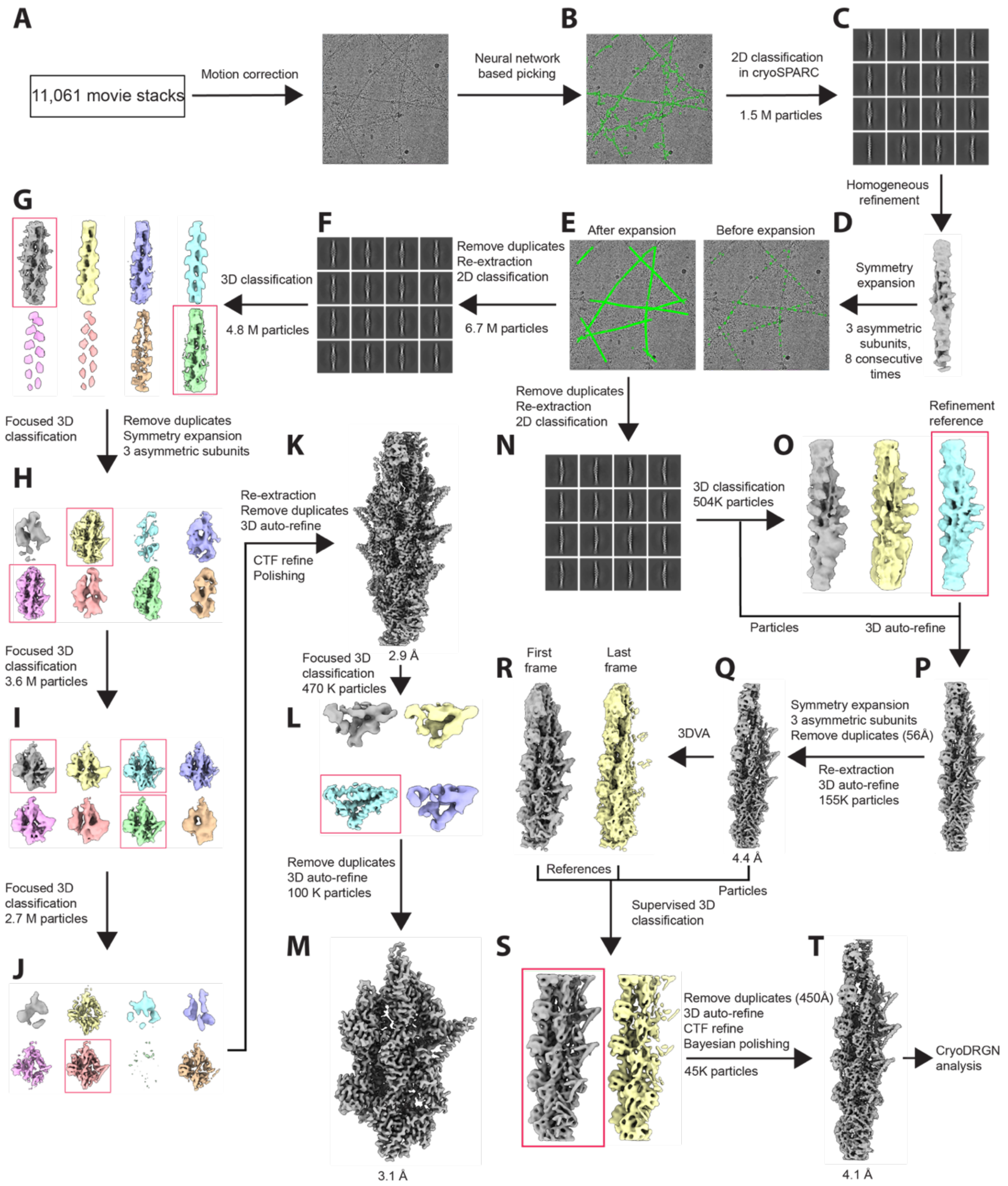

Fig. S3. Cryo-EM data processing workflow.

(**A-C**) Initial 2D processing, including motion correction of raw micrographs (**A**), neural-network based picking of heterogeneously decorated filaments (**B**), and reference-free 2D classification (**C**). (**D-E**) Strategy for fully capturing incompletely picked F-actin in micrographs, consisting of homogeneous refinement (**D**) followed by symmetry expansion using a helical twist of -167°, a helical rise of 27 Å, and 23 asymmetric subunits (**E**). (**F-M**) Workflow for obtaining a high-resolution reconstruction of the pentamer bound to straight F-actin, which includes:

selecting 2D classes featuring αE-catenin ABD and afadin-CC density on both strands of straight filaments (**F**); 3D classification using helical symmetry (**G**); focused 3D classification without image alignment using a mask covering 5 actin subunits, 2 αE-catenin ABDs, and 1 afadin-CC (**H**); a first (**I**) and second (**J**) round of focused 3D classification using a mask which covers 3 actin subunits, 2 αE-catenin ABDs, and 1 afadin-CC; 3D auto-refinement, CTF refinement, and Bayesian polishing to obtain an initial reconstruction with moderate quality density for flexible αE-catenin ABD elements (**K**); and focused 3D classification using a mask which covers the C-terminal region of αE-catenin ABD (densities corresponding to residues 871-906) and afadin-CC (**L**). A final 3D auto-refinement produced the reconstruction where the F-actin binding interface of the αE-catenin ABD and afadin were well-resolved (**M**). (**N-T**) Workflow for visualizing the pentamer's cooperative binding to bent F-actin, which includes: selecting 2D classes with αE-catenin ABD and afadin-CC bound along one strand of bent filaments (**N**); asymmetric 3D classification (**O**); initial 3D auto-refinement (**P**) followed by a second round after symmetry expansion along the long pitch helical path to maintain strand registry (helical twist of 26°, helical rise of 55 Å, and 3 asymmetric subunits, **Q**); and 3DVA variability analysis (**R**) which produced references which were used for supervised 3D classification (**S**). 3D auto-refinement, CTF refinement, and Bayesian polishing produced the final reconstruction (**T**), which was used as the input for CryoDRGN variability analysis.

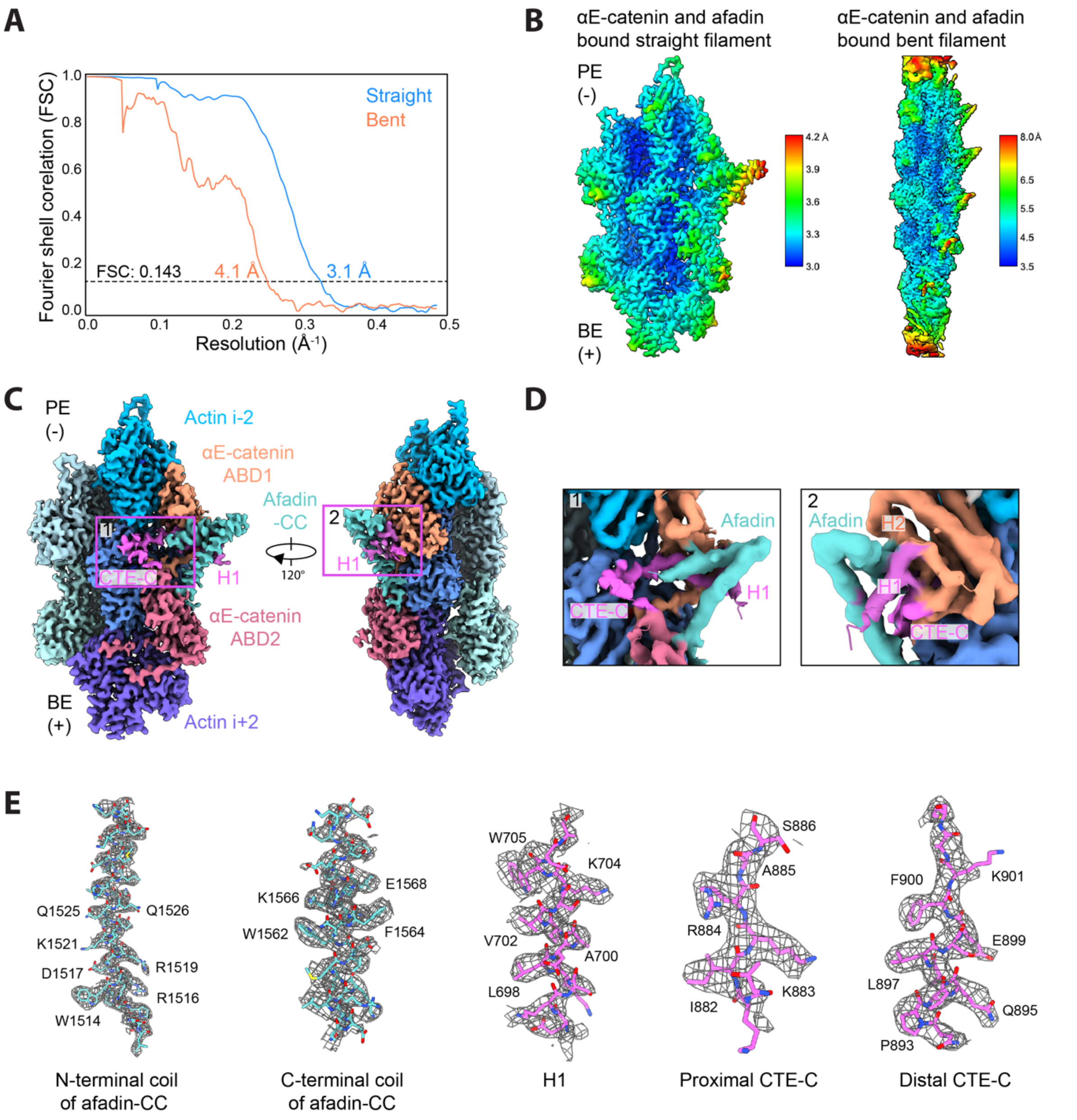

Fig. S4. Resolution assessment and density features of cryo-EM reconstructions.

(**A**) Gold-standard Fourier Shell Correlation (FSC) curves for the straight and curved pentamer-bound F-actin reconstructions. (**B**) Local resolution estimation of the two reconstructions. (**C**) Views of the straight pentamer-bound F-actin cryo-EM map, highlighting densities corresponding to flexible αE-catenin ABD segments stabilized by afadin-CC. (**D**) Detail views of boxed regions in C. The map was low-pass filtered to 6 Å to facilitate visualization. (**E**) Segmented cryo-EM map densities and atomic models of the afadin-CC and the αE-catenin structural elements it stabilizes.

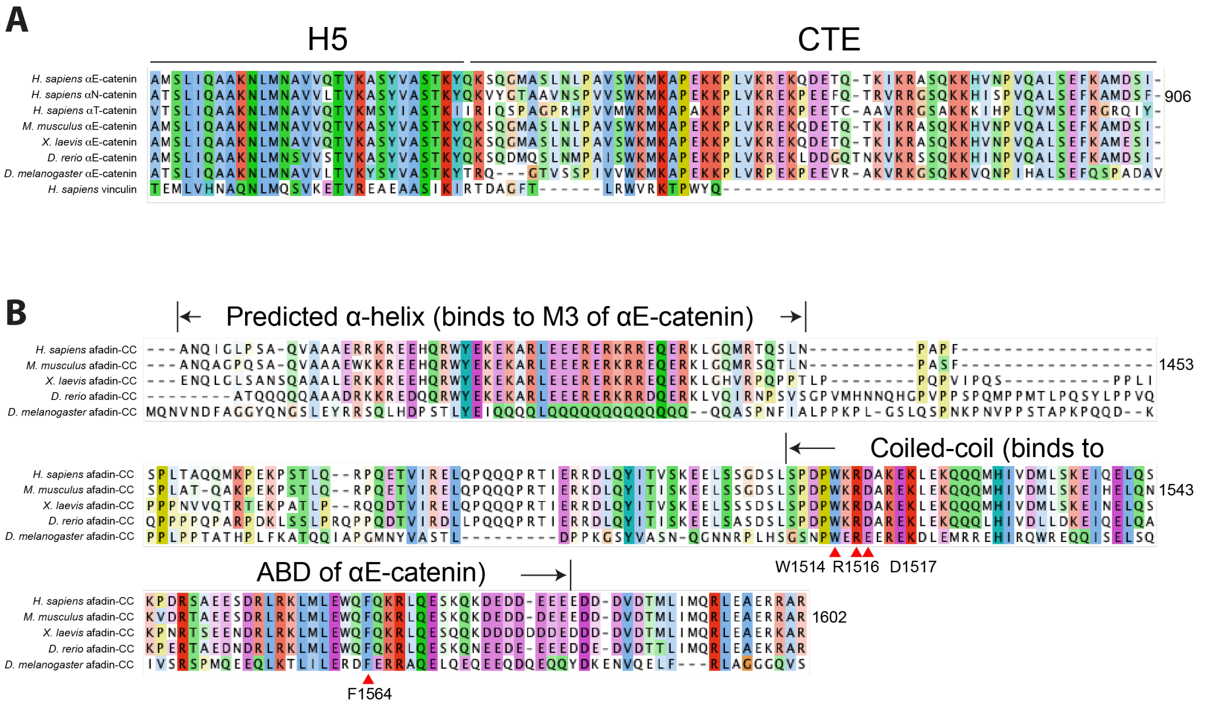

Fig. S5. Sequence alignments of the α-catenin CTE and afadin-CC.

(**A**) Sequence alignment of the α-catenin CTE among different species and isoforms. Sequences used for alignment are from *H. sapiens* αE-catenin (NP_001310911.1), *H. sapiens* αN-catenin (NP_004380.2), *H. sapiens* αT-catenin (NP_001120856.1), *M. musculus* αE-catenin (NP_033948.1), *X. laevis* αE-catenin (NP_001084100.1), *D. rerio* αE-catenin (NP_571531.1), *D. melanogaster* αE-catenin (NP_524219.1) and *H. sapiens* vinculin (NP_003364.1). (**B**) Sequence alignment of afadin's coiled-coil region. Aligned sequences are from *H. sapiens* afadin (NP_001353249.1), *M. musculus* afadin (NP_034936.1), *X. laevis* afadin (NP_001171575.1), *D. rerio* afadin (XP_021324351.1), and *D. melanogaster* canoe (NP_524232.2). Both alignments are colored by sequence conservation. Alignments were performed using Clustal Omega and visualized with Jalview.

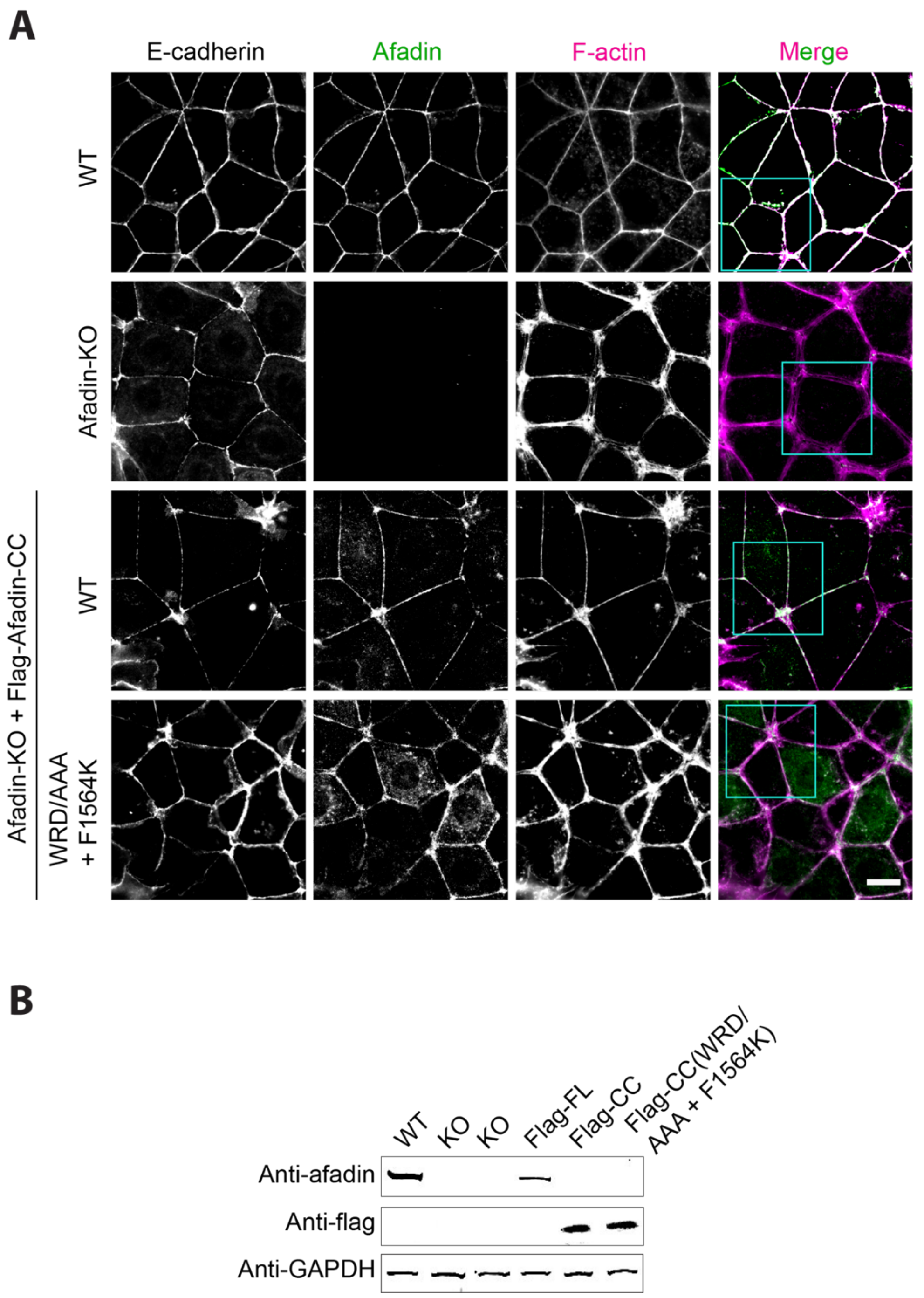

Fig. S6. Additional analysis of afadin-CC's role in proper actomyosin organization at AJs. (**A**) Expanded fields of view of the immunofluorescence images displayed in Figure 5A, visualizing multiple cells and AJs. Regions displayed in Figure 5A are indicated with boxes. Scale bar: 20 µm. (**B**) Western blots showing afadin knockout and overexpression efficiency in EpH4 cells.

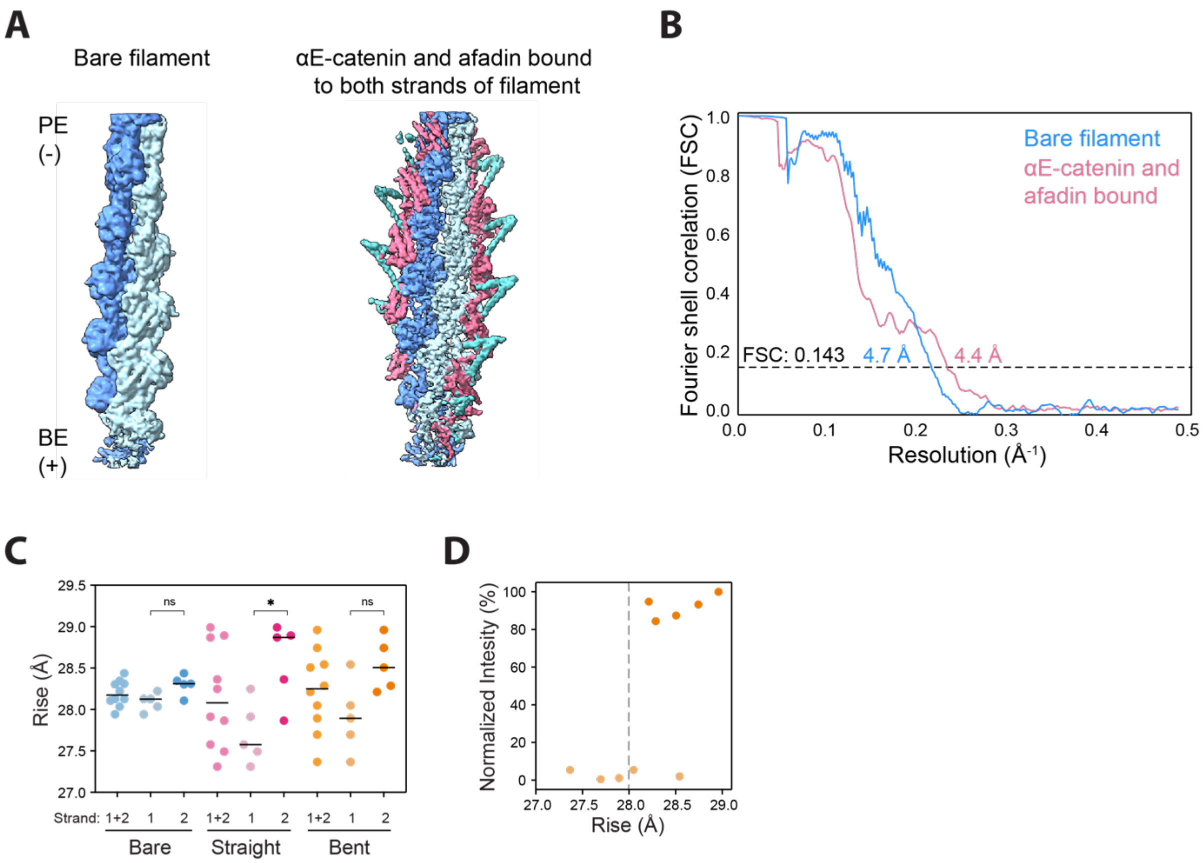

Fig. S7. Cryo-EM structures of bare F-actin and pentamer bound to both strands of F-actin spanning 13 actin subunits.

(**A**) 4.7 Å and 4.4 Å resolution cryo-EM density maps of bare F-actin (left) and pentamer bound to both strands of F-actin (right). (**B**) FSC curves for the bare F-actin and pentamer bound to both strands of F-actin reconstructions. (**C**) Quantification of the local rise of the bare filament ("bare"), straight filament with both strands decorated ("straight"), and the most curved filament (frame 9) from cryoDRGN analysis ("curved"). Circles represent the rise measured at each protomer index of the 10 central subunits. Strand 2 corresponds to the decorated strand of the curved filament. Bars represent means. Data were compared by one-way ANOVA with Tukey's multiple comparison test: NS, $p \geq 0.05$; *$p < 0.05$. (**D**) Quantification of complex intensity vs. local rise at each protomer index in the curved reconstruction from C. Vertical dashed line indicates rise of canonical straight F-actin.

Table S1. Cryo-EM data collection, refinement and validation statistics.

	F-actin binding interface of αE-catenin ABD (cadherin-catenin complex) and afadin (EMD-47194, PDB:9DVA)	αE-catenin ABD (cadherin-catenin complex) and afadin bound to bent F-actin (EMD-47195)	Bare F-actin (EMD-47196)	αE-catenin ABD (cadherin-catenin complex) and afadin bound to straight F-actin (EMD-47197)	Tomogram of the cadherin-catenin–vinculin-afadin complex bound to F-actin (EMD-47198)
**Data collection and processing**					
Collection modality	Single particle	Single particle	Single particle	Single particle	Tomography
Microscope	Titan Krios	Titan Krios	Titan Krios	Titan Krios	Titan Krios
Voltage (kV)	300	300	300	300	300
Detector	K2 Summit	K2 Summit	K2 Summit	K2 Summit	K3
Magnification	29,000	29,000	29,000	29,000	26,000
Electron exposure ($e^- / Å^2$)	61.26	61.26	61.26	61.26	107.82
Exposure rate (e^- / pixel / s)	1.53	1.53	1.53	1.53	30.0
Calibrated pixel size (Å)	1.03	1.03	1.03	1.03	2.60
Defocus range (µm)	–0.8 to –2.0	–0.8 to –2.0	–0.8 to –2.0	–0.8 to –2.0	–3.5
Symmetry imposed	C1	C1	C1	C1	-
Initial particle images (no.)	1,524,620	1,524,620	1,524,620	1,524,620	-
Final particle images (no.)	99,745	44,548	70676	14904	-
Map resolution (Å)	3.12	4.09	4.70	4.36	-
FSC threshold	0.143	0.143	0.143	0.143	-
**Refinement**					
Initial model (PDB ID)	6UPV	-	-	-	-
Model resolution (Å)	3.2	-	-	-	-
FSC threshold	0.5	-	-	-	-
Map sharpening B factor ($Å^2$)	–43.83	-57.92	-153.61	-97.14	-
Model composition	5 actin protomers,<br>2 αE-catenin,<br>1 afadin	-	-	-	-
Non-hydrogen atoms	18,529	-	-	-	-
Protein residues	2,354	-	-	-	-
Ligands	5 Mg.ADP	-	-	-	-
B factors ($Å^2$)					
Protein	81.72	-	-	-	-
Ligand	70.32	-	-	-	-
R.M.S. deviations					
Bond lengths (Å)	0.002	-	-	-	-
Bond angles (°)	0.504	-	-	-	-
**Validation**					
MolProbity score	1.19	-	-	-	-
Clash score	4.06	-	-	-	-
Poor rotamers (%)	0	-	-	-	-
Ramachandran plot					
Favored (%)	98.67	-	-	-	-
Allowed (%)	1.33	-	-	-	-
Disallowed (%)	0.00	-	-	-	-

Table S2. Constructs and primers

**Clones used for expression in FreeStyle 293-F cells**	**Primer 1**	**Primer 2**
pCAG-GFP-E-cadherin (734-844)	CCCGGGAGCTCCGGGGATCC CGGAGGAGAACGGTGGTC	AGCCGACGCGTCCGCTCGAG CTAGTCGTCCTCACCACCG
pCAG-GFP-Halo-E-cadherin (734-844)	CCCGGGAGCTCCGGGGATCC CGGAGGAGAACGGTGGTC	AGCCGACGCGTCCGCTCGAG CTAGTCGTCCTCACCACCG
pCAG-β-catenin	GGCAAAGAATTATCGATCC ATGGCTACTCAAGCTGACC	CTTGTCGAGCCGACGCGTCCG TTACAGGTCAGTATCAAACC
pCAG-α-catenin	GGCAAAGAATTATCGATCC ATGACTGCCGTCCACGCAGGCAAC	CTTGTCGAGCCGACGCGTCCG TCAGATGCTGTCCATGGC
pCAG-α-catenin (1-636)	GGCAAAGAATTATCGATCC ATGACTGCCGTCCACGCAGGCAAC	CTTGTCGAGCCGACGCGTCCG TTA CTCGGGGGTCCTGATCATC
pCAG-α-catenin (1-873)	GGCAAAGAATTATCGATCC ATGACTGCCGTCCACGCAGGCAAC	CTTGTCGAGCCGACGCGTCCG TCACTCTCTCTTCACCAACGG
pCAG-α-catenin (1-890)	GGCAAAGAATTATCGATCC ATGACTGCCGTCCACGCAGGCAAC	CTTGTCGAGCCGACGCGTCCG TCAGTGTTTCTTCTGAGAAG
pCAG-GFP-α-catenin (N1-2: 1-279)	CCCGGGAGCTCCGGGGATCC ATGACTGCCGTCCACGCAG	AGCCGACGCGTCCGCTCGAG TTATGCCAGCTCTCCGCCACTG
pCAG-GFP-α-catenin (M1: 262-395)	CCCGGGAGCTCCGGGGATCC ACTGCATCAGATGATGCTG	AGCCGACGCGTCCGCTCGAG TTACAGGAAAGAATCTGATACG
pCAG-GFP-α-catenin (M2: 396-507)	CCCGGGAGCTCCGGGGATCC GAGACCAATGTCCCTCTAT	AGCCGACGCGTCCGCTCGAG TTAGGAAGTAATGTCATCAAC
pCAG-GFP-α-catenin (M3: 506-636)	CCCGGGAGCTCCGGGGATCC ACTTCCATCGATGACTTC	AGCCGACGCGTCCGCTCGAG TTA CTCGGGGGTCCTGATCATC
pCAG-GFP-α-catenin (ABD: 636-906)	CCCGGGAGCTCCGGGGATCC ATCAGGACCCCCGAGGAG	AGCCGACGCGTCCGCTCGAG TCAGATGCTGTCCATGGC
pCAG-GFP-vinculin	CCCGGGAGCTCCGGGGATCC ATGCCAGTGTTTCATACGCGCAC	AGTCACGATGCGGCCGCTCGAG CTACTGGTACCAGGGAGTCTTTC
pCAG-Flag-vinculin	CCCGGGAGCTCCGGGGATCC ATGCCAGTGTTTCATACGCGCAC	AGTCACGATGCGGCCGCTCGAG CTACTGGTACCAGGGAGTCTTTC
pCAG-GFP-afadin-CC	CCCGGGAGCTCCGGGGATCC GCCAACCAGGCAGGACCCCAG	AGCCGACGCGTCCGCTCGAG TCACCTGGCTCTCCGCTCGGCCTC
pCAG-Flag-afadin-CC	CCCGGGAGCTCCGGGGATCC GCCAACCAGGCAGGACCCCAG	AGCCGACGCGTCCGCTCGAG TCACCTGGCTCTCCGCTCGGCCTC
pCAG-Flag-afadin-CC-N (1393-1510)	CCCGGGAGCTCCGGGGATCC GCCAACCAGGCAGGACCCCAG	AGCCGACGCGTCCGCTCGAG TCATGGAGACAGACTATCACC
pCAG-Flag-afadin-CC-C (1510-1602)	CCCGGGAGCTCCGGGGATCC TCTCCAGACCCCTGGAAACG	AGCCGACGCGTCCGCTCGAG TCACCTGGCTCTCCGCTCGGCCTC
**Clones used for lentiviral production**	**Primer 1**	**Primer 2**
pCDH-Flag-afadin	CAGCTAGAGCTAGCGAATTCGCCACC ATGGATTACAAGGATGACGATGACAA GATGTCGGCGGGCGGCCGCGACGAAG A	GATCCTTGCGGCCGCGGATCCTCACTT TGTGTTCAGTTCATTCTCG
pCDH-Flag-afadin-CC	CAGCTAGAGCTAGCGAATTCGCCACC ATGGATTACAAGGATGACGATGACAA GGCCAACCAGGCAGGACCCCAG	GATCCTTGCGGCCGCGGATCCTCACCT GGCTCTCCGCTCGGCCTC
**Primers used for generating point mutations**	**Primer 1**	**Primer 2**
Vinculin (T12: D974A/K975A/R976A/R978A)	CCAAGCAGTGCACAGCCGCAGCTATT GCCACCAACCTCTTACAGG	CCTGTAAGAGGTTGGTGGCAATAGCT GCGGCTGTGCACTGCTTGG
Vinculin (I997A/V1001A)	GCACCCAGCTCAAAGCCCTGTCCACA GCAAAGGCCACCATGCTG	CAGCATGGTGGCCTTTGCTGTGGACA GGGCTTTGAGCTGGGTGC
α-catenin (M319G)	GTGGGGCTGCCCTGGGTGCTGACTCAT CCTG	CAGGATGAGTCAGCACCCAGGGCAGC CCCAC
α-catenin (R326E)	CTCATCCTGCACAGAGGATGACCGTC GGG	CCCGACGGTCATCCTCTGTGCAGGAT GAG
α-catenin (R551E)	GAGGCCGGGCAGCCGAGGTCATTCAT GTAGTC	GACTACATGAATGACCTCGGCTGCCC GGCCTC
α-catenin (I882A/K883A/R884A)	GAGACGCAGACCAAGGCAGCAGCAGC TTCTCAGAAG	CTTCTGAGAAGCTGCTGCTGCCTTGGT CTGCGTCTC
Afadin (W1514A/R1516A/D1517A)	GTCTCCAGACCCCGCTAAAGCAGCTG CCAGGGAGAAG	CTTCTCCCTGGCAGCTGCTTTAGCGGG GTCTGGAGAC
Afadin (F1564K)	GGAGTGGCAGAAACAGAAGAGACTAC	GTAGTCTCTTCTGTTTCTGCCACTCC

Supplementary Movie Legends

Movie S1. Tomogram of the pentameric supra-complex binding along actin filaments.

Movie S2. Morph of the αE-catenin ABD model between the pre-bound and post-bound states. The αE-catenin ABD model in the pre-bound state is from the AlphaFold2 predicted structure of the full-length mouse αE-catenin (AF-P26231-F1), which contains the entire CTE-C.

Movie S3. Morph of the αE-catenin ABD model between the pre-bound and the afadin stabilized states. The same pre-bound model was used as in Video S2.
